# Supramolecular‐Engineered Bamboo Lignin Polymer with Enhanced Mechanical and Electrical Properties for Flexible Electronics

**DOI:** 10.1002/advs.202512983

**Published:** 2025-09-12

**Authors:** Zhimin Kou, Ye Sha, Lihong Hu, Meiting Liu, Fuchun Huang, Jie Wang, Caiying Bo, Yonghong Zhou, Dawei Zhao, Puyou Jia

**Affiliations:** ^1^ Institute of Chemical Industry of Forest Products Chinese Academy of Forestry (CAF) 16 Suojin North Road Nanjing 210042 China; ^2^ Department of Chemistry and Material Science College of Science Nanjing Forestry University Nanjing 210037 China; ^3^ Key Laboratory on Resources Chemicals and Materials of Ministry of Education Shenyang University of Chemical Technology Shenyang 110142 China

**Keywords:** disulfide bond, flexible sensors, functional ionogels, lignin, mechanical performances

## Abstract

Ionogels have emerged as a groundbreaking category of materials for flexible electronics, yet the challenge of integrating high mechanical stretchability, rapid electrical response, and reliable self‐repair in bio‐based ionogels persists. Here, a lignin‐derived ionogel is presented, designated P(LA‐TA)‐gel, through a straightforward, one‐step solvent‐free process. By leveraging arginine‐grafted lignin‐terminated polythioctic acid and incorporating imine and hydroxyl groups into the supramolecular framework, the P(LA‐TA)‐gel with both dynamic disulfide bonds and supramolecular hydrogen bonds achieves effective energy dissipation channels. This configuration adeptly balances mechanical stretchability—exhibiting a strain of 1233%—with dynamic reversibility, evidenced by an adhesion strength of 335.5 kPa. Notably, the P(LA‐TA)‐gel showcases rapid response characteristics, achieving a response time of 0.1 s, along with high ionic conductivity of 17.36 mS cm^−1^ and robust self‐healing capabilities exceeding 90%. When utilized in flexible sensors, this ionogel demonstrates a wide response range and high sensitivity. This study establishes a sustainable platform for the development of next‐generation flexible sensors and highlights significant potential applications across wearable electronics, electronic skin technology, and soft robotics.

## Graphical abstract

A lignin‐derived ionogel is developed through a straightforward, one‐step solvent‐free process. By leveraging arginine‐grafted lignin‐terminated polythioctic acid and incorporating imine and hydroxyl groups into the supramolecular framework. Its dynamic disulfide/hydrogen‐bond dual networks enable balanced stretchability‐reversibility while delivering adhesion, conductivity, and self‐healing. This molecularly engineered system establishes a sustainable platform for next‐generation flexible sensors in wearables, e‐skin, and soft robotics.

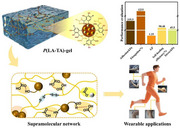

## 1 Introduction

In recent decades, flexible and stretchable electronics have made rapid advancements, spurred by their promising applications in soft robotics, human‐computer interaction, body motion detection, and health monitoring.^[^ ^1^, ^2^, ^3^, ^4^, ^5^ ^]^ Traditional sensors based on rigid electronic conductors, such as carbon and metals, hinder their ability to accommodate large deformations. In contrast, flexible and stretchable electronic devices offer significant advantages over traditional metal‐ or semiconductor‐based devices.^[^ ^3^, ^6^, ^7^ ^]^ Flexible strain sensors, which are non‐electrical measurement devices capable of detecting mechanical deformations and converting them into electrical signals, have gained prominence. Notably, continuous flexible strain sensors that allow for straightforward signal recording and processing hold substantial potential for next‐generation electronics.^[^ ^8^, ^9^, ^10^ ^]^ To overcome traditional sensor limitations, ionic gels are increasingly used as matrix materials due to their excellent deformability, conductivity, and skin‐like modulus.

Supramolecular‐interaction‐based ionic gels represent a promising approach for flexible sensor development, leveraging multivalent synergistic effects from interactions including hydrogen bonds,^[^ ^6^, ^11^, ^12^ ^]^ coordination bonds,^[^ ^13^, ^14^ ^]^ hydrophobic interactions,^[^ ^15^, ^16^ ^]^ van der Waals forces,^[^ ^17^, ^18^, ^19^ ^]^ π–π interactions,^[^ ^20^, ^21^ ^]^ and ionic bonds.^[^ ^22^, ^23^ ^]^ Supramolecular ionic gels typically consist of a supramolecular polymer network and an ionic liquid.^[^ ^24^, ^25^, ^26^ ^]^ Most ionic liquids are non‐volatile and stable in both air and water,^[^ ^27^, ^28^ ^]^ allowing supramolecular ionic gels to harness the benefits of ionic liquids, such as ionic conductivity and high stability, while encapsulating them within a polymer network. While this supramolecular network structure provides exceptional elasticity, flexibility, and stretchability to the ionic gel, it demonstrates a deficiency in self‐healing capabilities and reversible adhesion.^[^ ^29^, ^30^, ^31^, ^32^ ^]^ Thioctic acid (TA), featuring terminal disulfide bonds and carboxylic acid groups, addresses these limitations by contributing to essential supramolecular interactions for polymer formation.^[^ ^33^, ^34^, ^35^ ^]^ With a melting temperature of 70 °C, TA undergoes thermally initiated dynamic ring‐opening polymerization (ROP) of its five‐membered ring disulfide bond via hierarchical self‐assembly and dynamic disulfide exchange, generating a linear supramolecular polymer network.^[^ ^34^, ^36^ ^]^ This contrasts with conventional sensors reliant on elastomers blended with rigid conductive fillers, which require complex dispersion processes and suffer from extended healing times and limited conductivity due to filler rigidity.^[^ ^37^, ^38^, ^39^, ^40^ ^]^

To address these challenges, this study presents a novel lignin‐based ionic gel system crafted through a synergistic supramolecular design that integrates dynamic disulfide networks with non‐covalent interactions, including multiple hydrogen bonds, ionic bonds, and π–π stacking. Lignin, as nature's second most abundant renewable resource, offers a cost‐effective and abundant platform for ionogel production, significantly reducing manufacturing costs and enhancing economic viability.^[^ ^41^ ^]^ The incorporation of modified lignin, rich in reactive functional groups, enhances the system's chemical reactivity, facilitating the synthesis of high‐performance ionic gels. Lignin's phenolic hydroxyl groups act as effective free radical scavengers, providing an efficient capping agent for radical‐based materials. By embedding this modified lignin within ionic gel sensors, we achieve several advantages: 1) significantly improved mechanical and adhesive properties, 2) effective stabilization of polyTA to prevent self‐depolymerization, and 3) high‐value utilization of lignin. Concurrently, TA, with its terminal disulfide bonds and carboxylic acid groups, imparts dynamic reversibility to the ionic gel network. This dual dynamic strategy constructs a supramolecular architecture that enhances ion transport efficiency. Consequently, the resulting sensors exhibit superior electrical conductivity, rapid response times, efficient reusability, and accelerated self‐healing capabilities, effectively overcoming the limitations of previous materials. This innovative approach not only addresses the challenges mentioned earlier, but also contributes to the advancement of next‐generation flexible and stretchable electronic devices.

## 2 Results and Discussion

### 2.1 Design, Synthesis, and Characterizations of *P*(LA‐TA)‐gel

**Figure 1:**
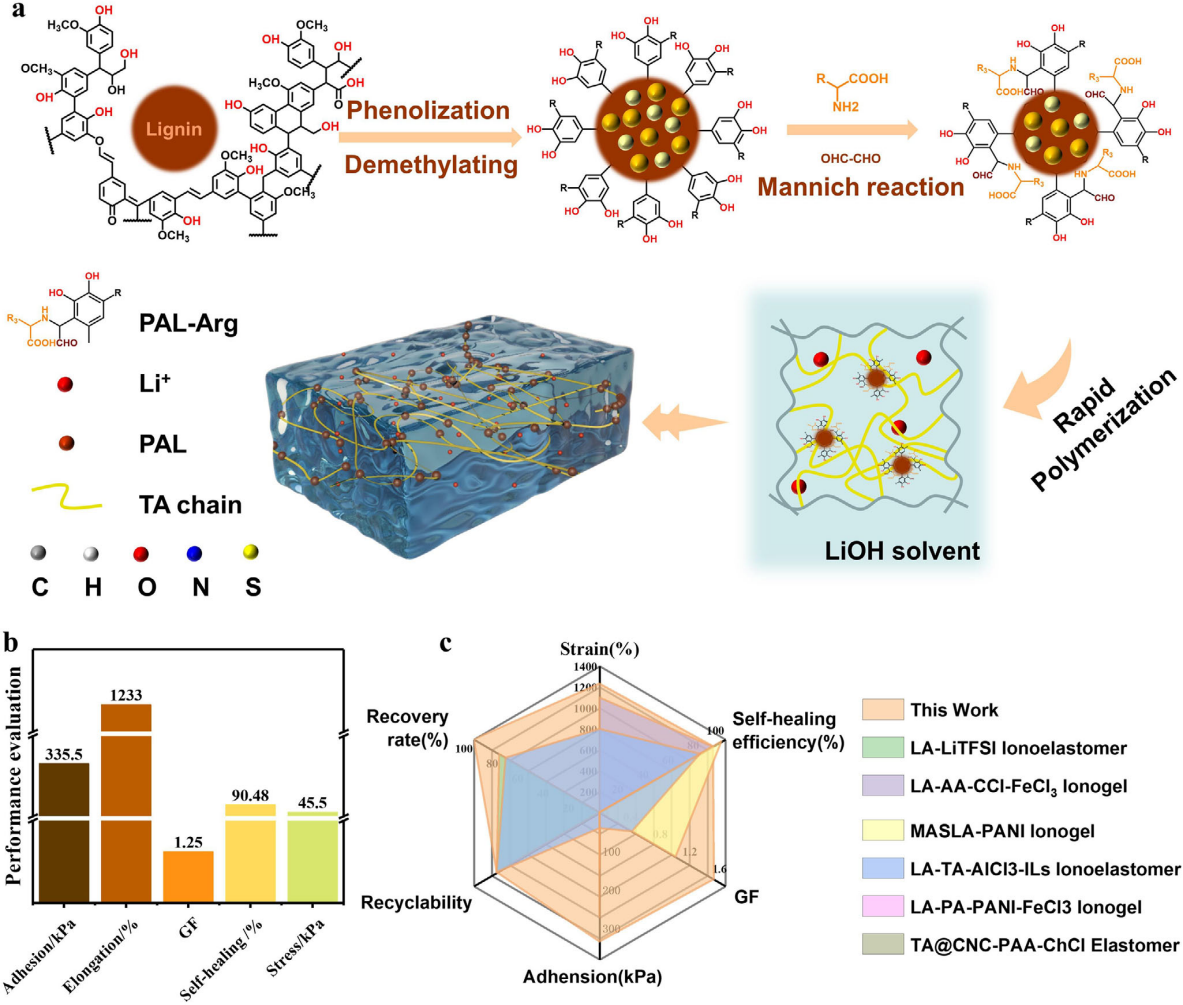
Design strategy of *P*(LA‐TA)‐gel. a) Schematic diagram of lignin demethylation/phenolization and synthesis of *P*(LA‐TA)‐gel. b) Performances of *P*(LA‐TA)‐gel. c) Comparison of the engineered *P*(LA‐TA)‐gel with the peer‐reported conductive composites.^[^ ^46^, ^47^, ^48^, ^49^, ^50^, ^51^ ^]^

**Figure 2:**
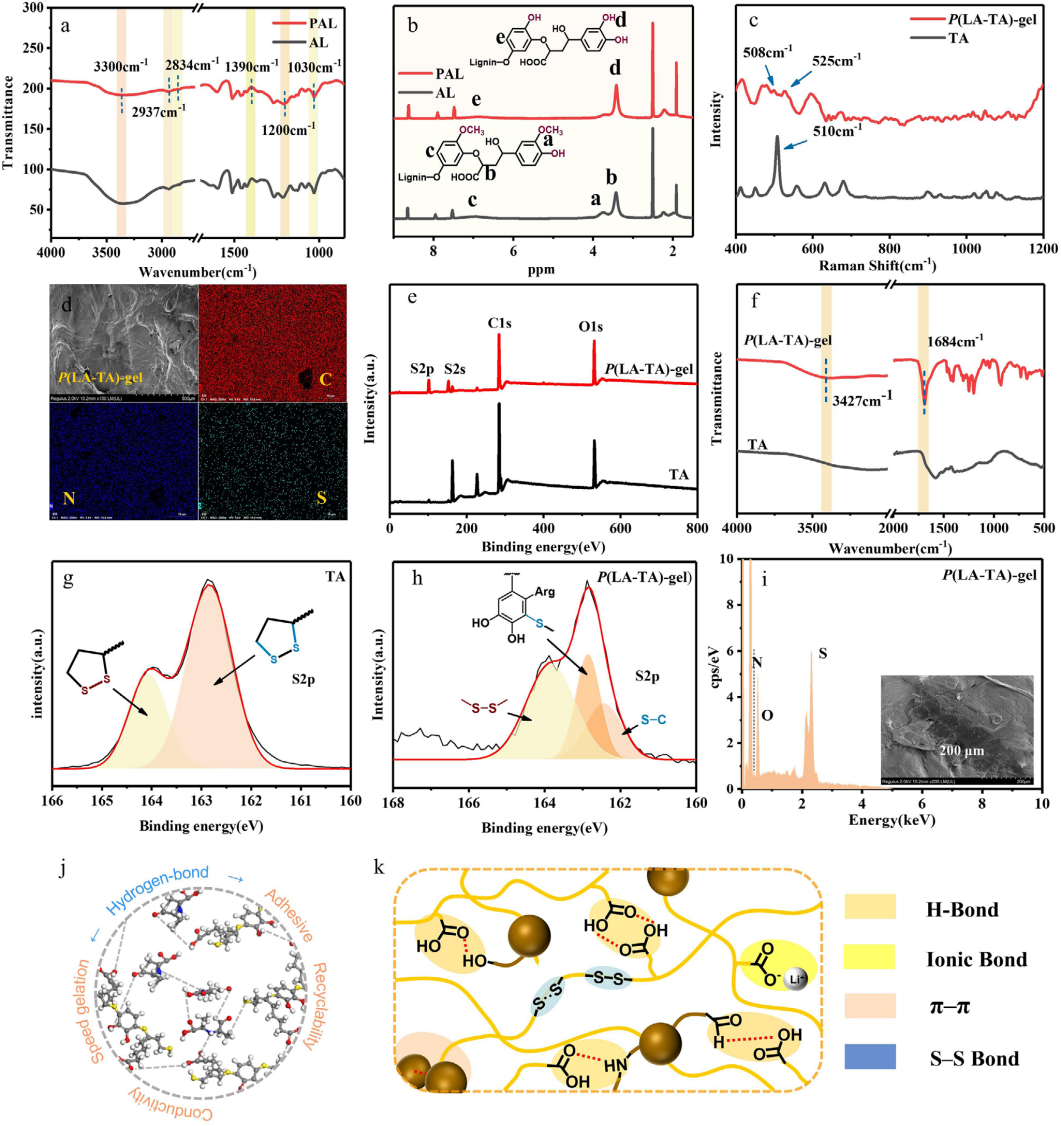
Design and investigate the structure of *P*(LA‐TA)‐gel. a) FTIR spectra of PAL and AL. b) Raman spectra of PAL and AL. c) Raman spectra of TA and *P*(LA‐TA)‐gel. d) SEM images of *P*(LA‐TA)‐gel after freeze‐drying and corresponding element mappings. e) XPS broad scan spectra of TA and *P*(LA‐TA)‐gel. f) FTIR spectra of TA and *P*(LA‐TA)‐gel; g) S2p XPS high‐resolution scanning spectra of TA. h) *P*(LA‐TA)‐gel S2p XPS high‐resolution spectra. i) EDS mapping of *P*(LA‐TA)‐gel. j) Supramolecular interactions (dynamic disulfide bond, multiple hydrogen bonds, ionic bonds, π–π stacking) existed in *P*(LA‐TA)‐gel. k) Schematic diagram of the supramolecular network of *P*(LA‐TA)‐gel.

Molecular modifying of lignin via acidic molten salt hydrate‐mediated demethylation/phenolation yielded pyrogallol‐modified lignin (PAL) (Figures S1–S8, Supporting Information), resolving inherent trade‐offs between mechanical robustness, self‐healing, conductivity, and adhesion in ionic gels (**Figure** **1**). Structural evolution was confirmed by Fourier transform infrared spectroscopy (FTIR, **Figure** **2**a) and ^1^H‐nuclear magnetic resonance (^1^H NMR, Figure 2b). FTIR analysis revealed no new absorption bands but varied absorption intensity after lignin modification. Relative to the 1510 cm^−1^ band, the intensities of methyl and methylene stretching vibrations (2937 and 2834 cm^−1^) decreased following modification, indicative of demethylation and ether bond cleavage. The modified lignin exhibited enhanced bands at 1200 cm^−1^ (phenolic hydroxyl) and 1030 cm^−1^ (alcoholic hydroxyl), along with a more intense and broadened hydroxyl absorption at 3300 cm^−1^, confirming successful introduction of additional polar hydroxyl groups into the lignin structure.^[^ ^33^, ^42^ ^]^ The ^1^H NMR spectral changes (Figure 2b) clearly demonstrated the chemical transformation, with the characteristic methoxy proton signal at 3.75 ppm diminishing after demethylation, while the emerging signal at 3.40 ppm evidenced the newly formed phenolic hydroxyl groups.^[^ ^43^ ^]^ Following demethylation and phenolation, the molecular weight distribution curve of lignin shifted slightly toward higher molecular weights (Figure S9, Supporting Information), suggesting a minor degree of condensation during the process. The increased molecular weight of modified lignin does not impede ion transport. This structural modification is advantageous for subsequent ionic gel preparation. Subsequent arginine functionalization produced PAL‐Arg (Figure S2, Supporting Information), which was integrated with thioctic acid (TA) prepolymerized under LiOH catalysis. Thermally initiated Arg‐mediated inverse vulcanization (Figure S3, Supporting Information),^[^ ^44^ ^]^ PAL‐Arg, with its polar amino and carboxyl groups, effectively quenched the chain‐end diradicals via inverse vulcanization. To systematically investigate the effect of lignin modification, a series of ionic gels with varying PAL‐Arg contents was prepared, designed as *P*(LA_x_‐TA)‐gel, where x represents the percentage of PAL‐Arg in the system.

The ring‐opening polymerization and subsequent quenching reaction were monitored using Raman and FTIR spectroscopy. In the Raman spectrum (Figure 2c), the characteristic disulfide vibration band at 510 cm^−1^ splits into two distinct peaks at 525 and 508 cm^−1^, confirming the ring‐opening polymerization of TA.^[^ ^45^ ^]^ Additionally, the emergence of a new peak at 673 cm^−1^, corresponding to C─S bond formation, demonstrated the successful quenching of polyTA's terminal diradicals by PAL‐Arg, ultimately yielding *P*(LA‐TA)‐gel.^[^ ^37^ ^]^ Its structure and micro‐morphology were further analyzed using scanning electron microscopy (SEM, Figure 2d) and X‐ray photoelectron spectroscopy (XPS, Figure 2e). Complementary FTIR analysis (Figure 2f) revealed further structural insights: the enhanced peak at 3427 cm^−1^ and the diminished peak at 1684 cm^−1^ collectively indicated the formation of hydrogen bonds and C─S bonds in the *P*(LA‐TA)‐gel network, consistent with cross‐linking through both non‐covalent and covalent interactions.

### 2.2 Design, Synthesis, and Characterizations of *P*(LA‐TA)‐Gel

To further confirm that the introduction of PAL‐Arg suppressed PTALi's thiol radicals and facilitated hydrogel formation, XPS was performed. As shown in Figure 2e, the XPS spectra of TA and *P*(LA‐TA)‐gel exhibited prominent peaks corresponding to C1s, O1s, S2s, and S2p. Deconvolution of the S2p spectra revealed distinct chemical states in both samples. In the S2p spectrum of TA (Figure 2g), two characteristic peaks were observed at 164.1 and 162.6 eV, corresponding to the S─S bond in the five‐membered ring and the C─S bond, respectively. In contrast, the S2p spectrum of *P*(LA‐TA)‐gel displayed an additional peak at 162.9 eV (Figure 2h), which was attributed to thiophenol. This result suggests that the catechol structure of PAL‐Arg effectively scavenges sulfur radicals in PTALi, corroborating the suppression of thiol radicals.

Morphological analysis of *P*(LA‐TA)‐gel revealed an interconnected and uniform microstructure (Figure 2d). To further verify the modification efficiency, energy‐dispersive X‐ray spectroscopy (EDS) mapping was employed to examine the elemental distribution in both lignin‐based monomers and freeze‐dried ionic gels. The results demonstrated a homogeneous dispersion of C, N, and S throughout *P*(LA‐TA)‐gel (Figure 2d), with increased N and S content (Figures S4–S8, Supporting Information), confirming successful modification. Additionally, quantitative elemental analysis of the ionic gel (Figure 2i) provided further evidence for the successful preparation of the material.

### 2.3 Regulated Mechanical Properties

The mechanical properties of *P*(LA‐TA)‐gel ionogels are synergistically governed by their hierarchical architecture: The abundant aromatic rings in lignin, along with the delocalized lithium ions within the network, facilitated strong π–π stacking and ionic interactions with the carboxyl groups of thioctic acid. These synergistic interactions significantly enhanced the cross‐linking density and mechanical properties of the resulting ionic gel. PAL's rigid aromatic framework provides structural integrity, while its functional moieties (phenolic hydroxyls, imine groups) enable supramolecular cross‐linking through hydrogen bonding and π‐π stacking.^[^ ^27^ ^]^ Concurrently, thioctic acid (TA) serves as a dynamic crosslinker via reversible disulfide bonds (─S─S─), with bond density and exchange kinetics dictating crosslinking density, modulus, and self‐healing efficiency. Under stress, reversible cleavage/reformation of these dynamic bonds facilitates energy dissipation, enhancing toughness and fatigue resistance. Precise modulation of lignin/TA ratios, reaction conditions, and ionic liquid selection allows tailored optimization of strength‐elasticity‐toughness‐healing trade‐offs.

**Figure 3:**
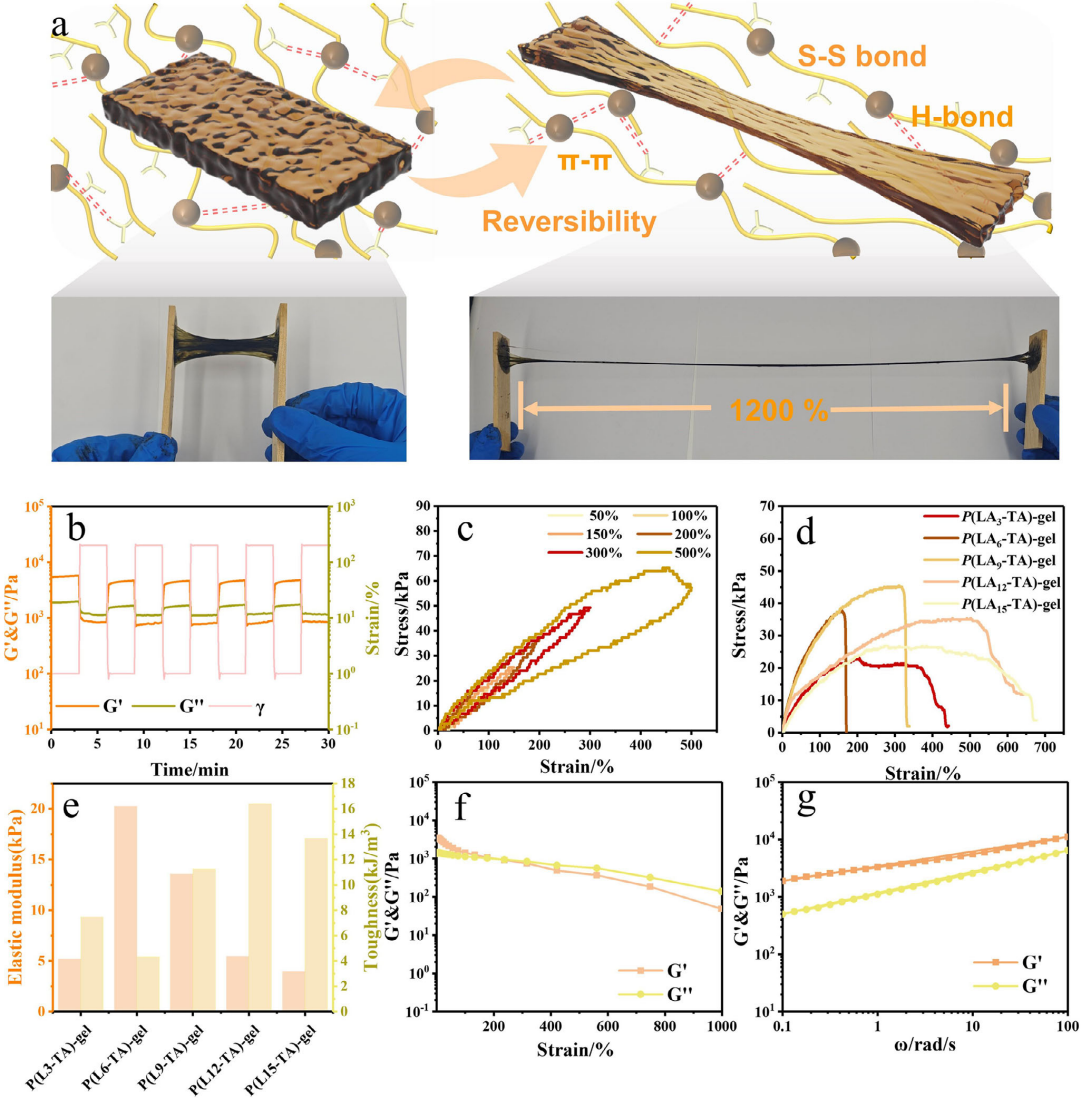
Investigating the design mechanism and physical properties of *P*(LA‐TA)‐gel. a) Tensile schematic diagram of *P*(LA‐TA)‐gel. b) Modulus change of *P*(LA‐TA)‐gel at 1% and 200% strain amplitude with a fixed frequency of 1.0 Hz and an interval of 3 min. c) Cyclic tensile curve of *P*(LA‐TA)‐gel. d) Tensile stress‐strain curve of *P*(LA‐TA)‐gel. e) Elastic modulus and toughness of the *P*(LA‐TA)‐gel. f) Modulus change of *P*(LA‐TA)‐gel with increased strain amplitude (0.1–1000%). g) Storage modulus (*G*') and loss modulus (*G*") of *P*(LA‐TA)‐gel versus frequency.

Rheological analysis confirms elastic dominance (*G*′ > *G*″ across frequencies) arising from molecular entanglement and cross‐linking.^[^ ^52^ ^]^ while exceptional extensibility (>1200% strain, **Figures** **3**a; S10 and S11, Supporting Information) demonstrates fracture resistance (Figure 3b). Critically, cyclic tests reveal minimal hysteresis with <10% residual strain at 500% deformation (Figures 3c,d; S12, Supporting Information), attributable to energy dissipation through reversible hydrogen bond/disulfide cleavage and subsequent reformation. Enhanced modulus/toughness (Figure 3e) stems from lignin‐induced physical entanglement and hydrogen bonding that facilitate stress transfer. This dynamic bond‐mediated recovery establishes structural equilibrium during prolonged cycling, where diminishing energy dissipation converges with stabilized residual strain.

### 2.4 Adhesive Performance and Reusability

This gel system constructs a 3D network structure with hierarchical dynamic bonding characteristics through the synergistic interplay of the following critical terms: 1) the multiple hydrogen‐bonding networks formed between the abundant phenolic hydroxyl groups and imine bonds within PAL, and TA molecules; and 2) the dynamic covalent crosslinking action mediated by the dynamic covalent disulfide bonds (─S─S─) of TA. The polyphenolic structure of lignin provides the gel with excellent interfacial wettability and adhesive motifs. This allows the gel to firmly anchor onto diverse substrates through hydrogen bonding, π–π stacking, and other non‐covalent interactions. Concurrently, the dynamic covalent crosslinking formed by lipoic acid dissipates energy through bond scission and recombination, which significantly enhanced the gel's cohesive strength, toughness, and inhibited interfacial debonding.^[^ ^48^ ^]^

**Figure 4:**
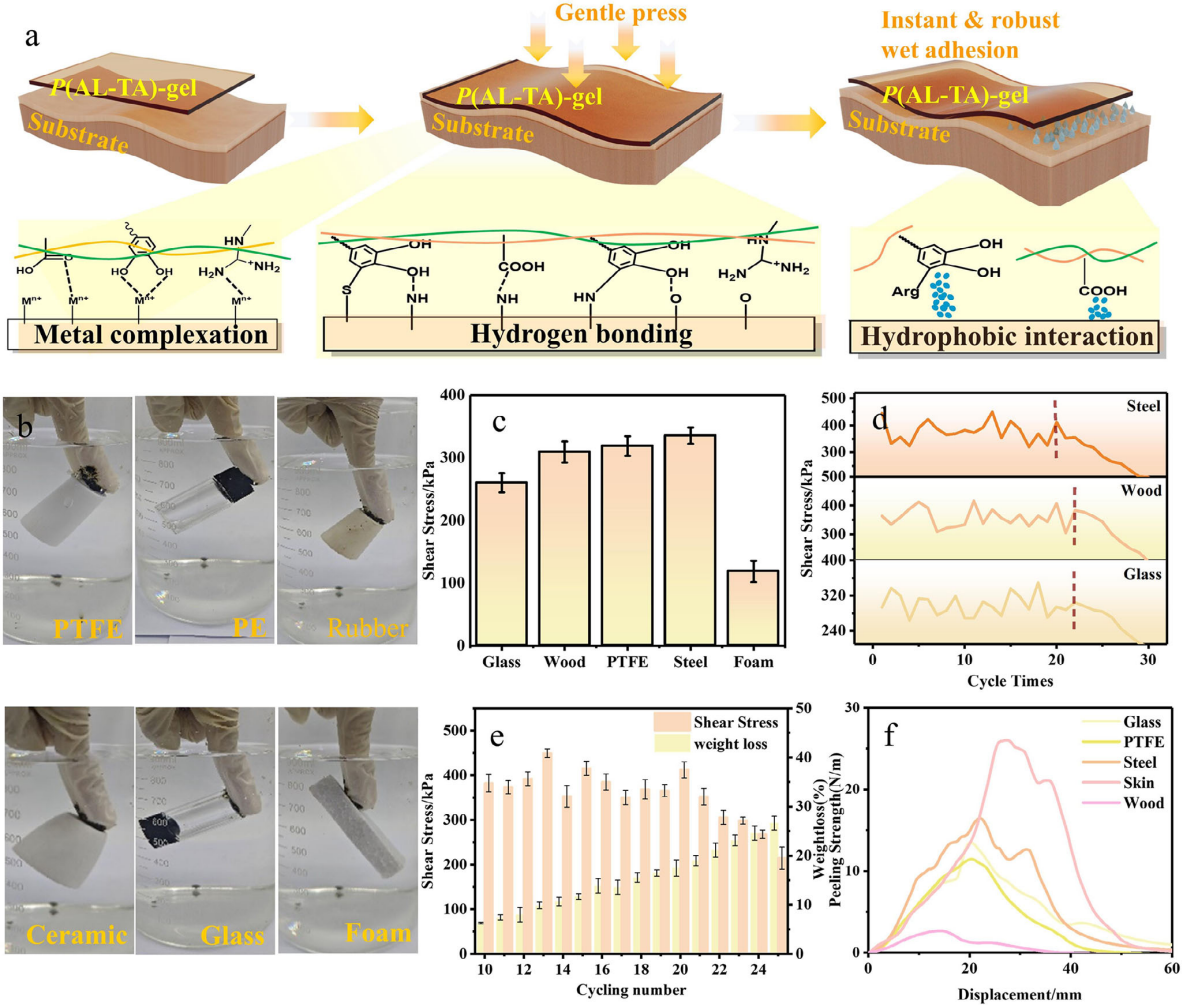
Investigating the thermal stability and adhesion of *P*(LA‐TA)‐gel. a) Schematic diagram of the adhesion for *P*(LA‐TA)‐gel to different substrate surfaces. b) Underwater adhesion of *P*(LA‐TA)‐gel to different substrates. c) Shear strength of *P*(LA‐TA)‐gel on different substrate surfaces. d) Variation of the bonding strength of *P*(LA‐TA)‐gel over 30 reversible bonding cycles. e) Variation of the bond strength of *P*(LA‐TA)‐gel with mass loss over 25 reversible bonding cycles. f) Load–displacement variation curves during the adhesion strength testing process.

Furthermore, the introduction of an ionic liquid medium not only promotes molecular diffusion and interfacial compatibility but also synergistically regulates the gel's rheological behavior and optimizes stress distribution through its inherent viscoelasticity.^[^ ^49^ ^]^ The adhesive strength of the *P*(LA‐TA)‐gel (e.g., shear and peel) is critically dependent on structural parameters, including network crosslinking density and the proportion of dynamic bonds. The recent reports reveal that moderate crosslinking optimizes the balance between cohesive energy and interfacial adhesion, while excessive crosslinking increases brittleness.^[^ ^23^, ^53^ ^]^ Dynamic bonds impart thermoreversibility and enable exceptional reusability (>20 cycles), although performance declines beyond this point due to cumulative mass loss. Unique interfacial structures formed on hydrophilic and metallic substrates via metal chelation and hydrogen bonding (**Figures** **4**a; S13, Supporting Information) significantly enhance adhesion, achieving a shear strength of 335.50 kPa (Figures 4b,c; S14, Supporting Information). Hydrophobic chains and aromatic rings of *P*(LA‐TA)‐gel confer water resistance and preserve adhesive performance under humid conditions (Figures 4b; S15–S17, Supporting Information), while a low swelling rate ensures dimensional stability in high humidity (Figure S18, Supporting Information).

Compared to existing conductive ionic gels and composite elastomers (Figure 1c; Table S1, Supporting Information), *P*(LA‐TA)‐gel exhibits superior rapid response, adhesion strength, self‐healing efficiency, and sensing sensitivity (Figure 4d–f). These enhanced properties originate from the synergistic interplay of supramolecular interactions and dynamic covalent bonds, enabling directional optimization through precise modulation of the lignin/lipoic acid ratio, ionic liquid type, and crosslinking conditions for high‐performance, intelligent‐responsive adhesion.

### 2.5 Room‐Temperature Self‐Healing Performance and Recyclability

The abundant benzene rings in *P*(LA‐TA)‐gel form supramolecular interactions through strong non‐covalent bonds, including multiple hydrogen bonds and π–π stacking. These interactions synergize with reversible dynamic disulfide bonds to significantly enhance the material's self‐healing efficiency. Concurrently, the flexible alkyl chains derived from TA improve ductility via entropy‐driven mechanisms. Elevated temperatures promote segmental motion, while the cleavage of dynamic bonds after material damage increases local entropy. This process facilitates molecular chain rearrangement into thermodynamically stable configurations. Furthermore, the hydrogen‐bonding network dynamically modulates *P*(LA‐TA)‐gel's viscoelasticity. This results in a reversibly crosslinked network that exhibits exceptional self‐healing performance and recyclability.

**Figure 5:**
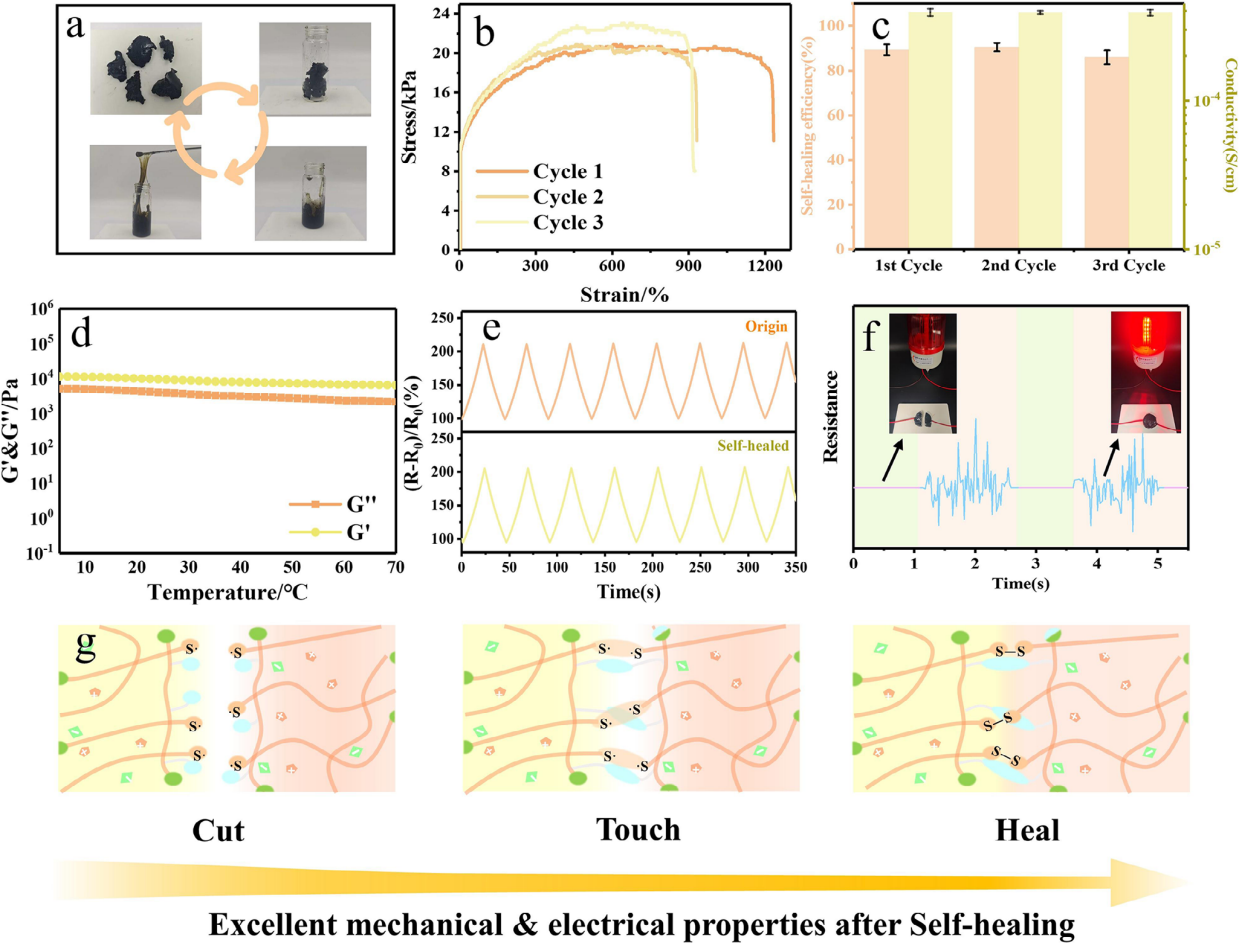
Investigating the thermal stability and self‐healing of *P*(LA‐TA)‐gel. a) Schematic diagram of cyclic processing of *P*(LA‐TA)‐gel at 80 °C for 15 min. b) Stress‐strain curve of *P*(LA‐TA)‐gel after multiple repairs. c) Self‐healing efficiency and conductivity of *P*(LA‐TA)‐gel. d) Modulus change of *P*(LA‐TA)‐gel at increasing temperature (0–70 °C). e) Electrical signal of *P*(LA‐TA)‐gel before and after self‐healing. f) electrical signals (under 70% strain) of *P*(LA‐TA)‐gel before and after complete cutting and self‐healing. g) Schematic representation of the self‐healing mechanism of the *P*(LA‐TA)‐gel.

*P*(LA‐TA)‐gel exhibits remarkable self‐healing properties driven by dynamic disulfide bonds, enabling reversible cleavage, reformation, and molecular chain reorganization (**Figure** **5**a). Upon damage, initial interfacial healing occurs via hydrogen bonding, which subsequently strengthens through disulfide bond exchange, fully restoring mechanical properties without external stimuli or healing agents.^[^ ^54^, ^55^ ^]^ The healed gel achieves a tensile strength of 23.1 kPa with improved deformation characteristics (Figures 5b; S19, Supporting Information). Although ionic transport channels are disrupted during damage, conductivity recovers rapidly (≈90% recovery rate) upon reconnection (Figure 5c). Structural stability (G' > G) is maintained below 70 °C (Figure 5d), while heating to 80 °C dissociates disulfide bonds, inducing a gel‐to‐sol transition. Crucially, cooling reforms the original gel, highlighting *P*(LA‐TA)‐gel's excellent recyclability. Cyclic resistance monitoring confirmed stable sensing performance under normal operation and immediate recovery (<0.4 s) to baseline resistance after self‐healing (Figure 5e,f), collectively demonstrating outstanding electrical self‐healing and cyclic processing capability (Figure 5g).

Rheological analysis revealed the relationship between the structure of *P*(LA‐TA)‐gel and its dynamic reversibility. As shown in Figure 3f, the storage modulus (*G*′) initially exceeded the loss modulus (*G*″) at strains below 206%, confirming the structural stability of the ionic gel. However, when the strain surpassed 206%, G″ became dominant, leading to the breakdown of the *P*(LA‐TA)‐gel network and a transition from a gel to a sol state. To further investigate its self‐healing capability, the strain was cyclically alternated between 1% and 200% (3 min per step). Under 200% strain, G′ decreased (Figure 3b), indicating disruption of the hydrogel network. Yet, upon returning to 1% strain, the hydrogel rapidly recovered its original structure, with G′ and G″ returning to their initial values—demonstrating exceptional self‐healing performance. Moreover, this recovery remained reversible over multiple cycles (Figure 5b), proving that *P*(LA‐TA)‐gel can repeatedly self‐heal even after mechanical damage. These findings highlight how the dynamic bonds within the *P*(LA‐TA)‐gel network govern its reversible structural behavior.

### 2.6 Electrical Sensing Properties

**Figure 6:**
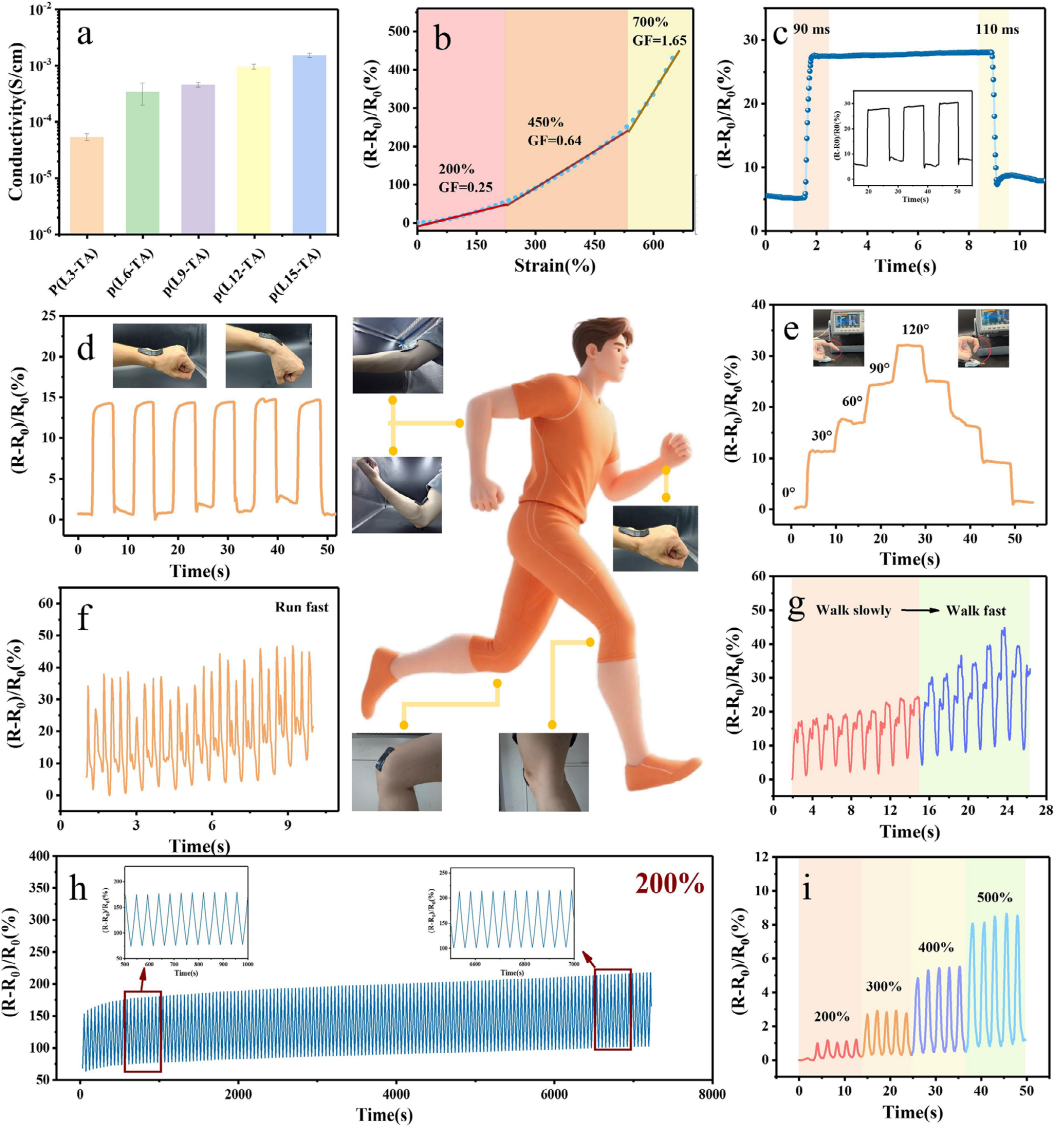
Investigating the electronic performances of *P*(LA‐TA)‐gel. a) Conductivity of different *P*(LA‐TA)‐gel. b) The gauge factors of *P*(LA‐TA)‐gel strain sensors in different strain ranges. c) Rapid response of electrical signals under 100% strain. The *P*(LA‐TA)‐gel flexible sensor detects the relative resistance change response of various human movements on the surface of human skin. d) bending the wrist multiple times. e) bending the finger at different angles. f) at the ankle during running. g) walking fast and slowly. h) The stable resistance changes rate of the *P*(LA‐TA)‐gel flexible sensor for 600 stretch cycles at 200% strain. i) The change in resistance of *P*(LA‐TA)‐gel at different strains.

The 3D dynamic network structure of *P*(LA‐TA)‐gel exhibits an intrinsic correlation with its electrosensing performance. This system achieves signal transduction through the synergistic interaction between an ionic liquid‐mediated continuous conductive phase and a dynamically bonded network: the ionic liquid provides mobile ionic carriers, whose concentration and mobility directly determine the intrinsic conductivity of the gel (1.52 ± 0.12 mS·cm^−1^) (**Figure** **6**a), ions migrate through the hydrogen‐bonded network via direct interactions with hydrogen bonds. These interactions lower ion migration energy barriers, facilitating ion mobility within the gel. The polyphenolic framework of modified lignin promotes electron delocalization via π‐π conjugation, while the reconfigurable crosslinking points formed by dynamic disulfide bonds (─S─S─) in lipoic acid and multiple hydrogen bonds within the network architecture endow the system with adaptability. This structure provides mechanical integrity and stability while establishing relatively stable ion‐conduction pathways.^[^ ^56^ ^]^ Under external stimuli (strain/temperature/chemical species), the dynamic reorganization of the network topology enables sensitive resistive/capacitive responses by modulating ion transport channel dimensions and carrier density.

Quantitative strain‐resistance analysis (Figure 6b) reveals a progressive relative resistance increase with applied force, demonstrating high strain sensitivity across tested ranges (gauge factors: GF = 0.25 at 0–200%, GF = 0.64 at 200–500%, GF = 1.65 at 500–700% strain). *P*(LA‐TA)‐gel exhibits ultrafast response (≈0.1s) and recovery (≈0.1s) capabilities, maintaining stable resistance during constant strain and complete structural/electrical restoration upon stress release (Figure 6c).

This synergy of stretchability, toughness, self‐healing, and stable conductivity enables efficient mechanoelectrical transduction for wearable sensing. When mounted on joints (Figure 6d,e), the sensor achieves real‐time monitoring of human motion through rapid, distinguishable resistance changes corresponding to joint angles and strain levels during activities (Figure 6f,g; Figure S20, Supporting Information). It maintains signal stability at fixed positions while responding sensitively to movement, attributed to strain‐modulated ion diffusion pathways. Robust durability is confirmed through 600 stretching cycles at 200% strain (Figure 6h) and consistent conductivity across varying strains (Figure 6i), with dynamic reconstruction properties ensuring reliable long‐term performance for wearable applications.

## 3 Conclusion

This study demonstrates a solvent‐free strategy for fabricating supramolecular‐engineered lignin ionogels that circumvent conventional metal salt/quencher dependencies. By arginine‐grafted lignin‐terminated polyTA networks with imine/hydroxyl functionalization, we established hierarchical hydrogen‐bonded architectures reinforced by dynamic disulfide bonds. The resultant material exhibits multifunctionality: extreme elasticity (≈1200% strain) coupled with ultrafast response/recovery kinetics of only 0.1 s, self‐healing efficiency of over 90%, and robust self‐adhesion of 335.50 kPa. Strong electro‐mechanical coupling with ionic liquids confers dual strain/temperature sensitivity, while the dynamic covalent‐supramolecular synergy provides tunable mechanoelectrical properties. These collective advances position *P*(LA‐TA)‐gel as transformative platforms for next‐generation wearable biosensors, durable soft electronics, and interactive human‐machine interfaces.

## 4 Experimental Section

### Materials

Alkali lignin (AL) was obtained from Yibin Paper Co. Ltd. Pyrogallol (PG, AR, 99%) was purchased from Aladdin. Arginine (Arg, 99%) was purchased from Aladdin. Lithium chloride (LiCl, anhydrous, 99%) was purchased from Rhawn. Thioctic acid (TA, 99 %) was purchased from Aladdin. Lithium hydroxide (LiOH, anhydrous, 98 %), sodium hydroxide (NaOH, 99%), and Glyoxal solution (40%) were purchased from Aladdin. Ethyl Alcohol (95%) was purchased from Rhawn. All reagents were used as received, unless otherwise specified. Deionized water was used throughout the experiments.

### Synthesis of PAL

A mixture of 3.0 g alkaline lignin (AL) and 1.0 g phloroglucinol was suspended in 30 mL of an aqueous solution containing 63% LiCl and 2.4 mol L^−1^ HCl. The reaction was conducted at 110 °C for 2 h under vigorous stirring. Upon completion, the vial was immediately removed from the oil bath and quenched in an ice‐water bath to terminate the reaction. The solid product was collected by filtration, washed repeatedly with a 5% ethanol solution to remove unreacted phloroglucinol, and vacuum‐dried at 40 °C for 48 h to yield demethylated and phenolated lignin (PAL). The yield of PAL was 82.48%.

### Synthesis of PAL‐Arg

1.5 g of PAL and 1.2 g of arginine (Arg) were dispersed in 30 mL of sodium hydroxide aqueous solution, and the solution pH was adjusted to 11. The solution was stirred in an N_2_ atmosphere at 60 °C, and a 10% glyoxal solution was added. After 4 h of reaction, the mixture was cooled to room temperature, the pH was adjusted to 7–7.5, and centrifuged at 10 000 rpm for 10 min. The upper layer solution was collected. Following dialysis in 1000 Da bags for 3 days, the solution was dried, resulting in the formation of arginine grafted demethylated and phenolated lignin (PAL‐Arg). The yield of PAL‐Arg was 68.39%.

### Synthesis of *P*(LA‐TA)‐Gel

TA was introduced to the LiOH solution and heated at 75 °C under stirring for 5 min to obtain a homogeneous PTALi solution. Subsequently, PAL‐Arg were added to the PTALi solution and poured into the mold after heating for 10 min. After cooling the mixture to 25 °C, a *P*(LA‐TA)‐gel hydrogel was obtained. The detailed compositions of the samples are listed in Table S2 (Supporting Information).

## Conflict of Interest

The authors declare no conflict of interest.

## Author Contributions

L.H., P.J., and Y.Z. supervised the project. Z.K. carried out most of the experiments. Z.K., M.L., and F.H. participated in electrochemical tests and microscopic morphology analysis. Z.K. and Y.S. wrote the paper. L.H., J.W., and C.B. discussed the results and designed the mechanism. L.H., D.Z., and P.J. collectively reviewed the paper. All authors have read and approved the final submitted manuscript.

## Supporting information



Supporting Information

## Data Availability

The data that support the findings of this study are available from the corresponding author upon reasonable request.

## References

[1] H. Jin , M. O. G. Nayeem , S. Lee , N. Matsuhisa , D. Inoue , T. Yokota , D. Hashizume , T. Someya , ACS Nano. 2019, 13, 7905. 31244040 10.1021/acsnano.9b02297

[2] Y. Ma , Y. Zhang , S. Cai , Z. Han , X. Liu , F. Wang , Y. Cao , Z. Wang , H. Li , Y. Chen , X. Feng , Adv Mater 2020, 32, 1902062. 10.1002/adma.201902062 31243834

[3] Y. Yao , Z. Fan , X. Gong , D. Li , W. Yang , K. C. Leung , X. Wang , S. Liu , J. Yang , S. Xuan , Adv Funct Mater 2025, 2500572.

[4] H. Li , Y. Ma , Z. Liang , Z. Wang , Y. Cao , Y. Xu , H. Zhou , B. Lu , Y. Chen , Z. Han , S. Cai , X. Feng , Natl. Sci. Rev. 2020, 7, 849. 34692108 10.1093/nsr/nwaa022 PMC8288864

[5] X. Guo , S. Zhang , S. Patel , X. Sun , Y. Zhu , Z. Wei , R. Wang , X. He , Z. Wang , C. Yu , S. C. Tan , Sci. Adv. 2025, 11, adv8523. 10.1126/sciadv.adv8523 PMC12083530 40378220

[6] Q. Long , G. Jiang , J. Zhou , D. Zhao , H. Yu , Research 2024, 7, 0533. 39559347 10.34133/research.0533 PMC11570788

[7] G. Jiang , G. Wang , Y. Zhu , W. Cheng , K. Cao , G. Xu , D. Zhao , H. Yu , Research 2022, 2022, 9814767. 35711672 10.34133/2022/9814767 PMC9188022

[8] J. Liu , C. Jiang , Q. Yu , Y. Ni , C. Yu , W. Xu , Nat. Commun. 2025, 16, 756. 39824840 10.1038/s41467-024-55670-4 PMC11742687

[9] P. Cao , J. Wei , T. Zhang , H. Deng , Y. Han , Z. Chen , Y. Chen , Y. Guo , C. Ma , Adv Funct Mater 2025, 2501131.

[10] H. Jiang , R. Bai , Y. Zhao , S. Shi , G. Jiang , D. Zhao , Adv Funct Mater 2025, 2503512.

[11] G. Jiang , G. Xu , Q. Xia , H. Yu , D. Zhao , Small 2025, 21 , 2412538. 10.1002/smll.202412538 40190210

[12] X. Li , H. Jiang , Y. Zhang , Q. Long , G. Jiang , S. Zeng , J. Zhou , D. Zhao , Adv Funct Mater 2024, 34, 2408160.

[13] Y. Zhang , C. Cai , F. Li , S. Dong , Small 2023, 19, 2300857. 10.1002/smll.202300857 37035948

[14] Y. Li , F. Huang , P. J. Stang , S. Yin , Acc. Chem. Res. 2024, 57, 1174. 38557015 10.1021/acs.accounts.4c00031

[15] M. Yan , S. Wu , Y. Wang , M. Liang , M. Wang , W. Hu , G. Yu , Z. Mao , F. Huang , J. Zhou , Adv Mater 2024, 36, 2304249. 10.1002/adma.202304249 37478832

[16] L. Zhang , Q. Lei , M. Yi , Z. Zhang , X. Lian , J. Xu , S. Zhang , L. Li , B. Li , X. Bu , Angew. Chem. Int. Edit. 2025, 64, 202421753. 10.1002/anie.202421753 39548883

[17] S. Tokuda , S. Furukawa , Nat. Chem. 2025, 17, 672. 40102670 10.1038/s41557-025-01777-0

[18] P. Chen , K. Fu , X. Zhang , S. Zhao , H. Wu , G. Liu , Nano Res. 2024, 18, 94907095.

[19] S. Yue , G. Xiao , Q. Shi , X. Mu , M. Zhang , M. Xie , J. Cao , Adv Funct Mater 2025, 2503631.

[20] J. Mei , S. Lai , Y. Gong , W. Shi , J. Deng , T. Lu , D. Zhong , Angew. Chem. Int. Edit. 2025, 64, 202413413. 10.1002/anie.202413413 39243218

[21] Y. Haketa , K. Yamasumi , H. Maeda , Chem. Soc. Rev. 2023, 52, 7170. 37795542 10.1039/d3cs00581j

[22] L. Liu , Y. Deng , D. Qu , B. L. Feringa , H. Tian , Q. Zhang , Angew. Chem. Int. Edit. 2025, 64, 202424147. 10.1002/anie.202424147 39808487

[23] Q. Deng , S. Han , Y. Wu , Y. Chen , Y. Zhang , Y. Zhao , S. Chen , J. Zhu , Angew. Chem. Int. Edit. 2025, 64, 202415386. 10.1002/anie.202415386 39450609

[24] H. Wang , H. Wang , D. Chen , X. Tian , J. Yang , W. Liu , Small 2025, 21, 2501737. 10.1002/smll.202501737 40411837

[25] X. Lyu , H. Zhang , S. Shen , Y. Gong , P. Zhou , Z. Zou , Adv Mater 2024, 36, 2410572. 10.1002/adma.202410572 39292213

[26] Y. Gu , C. Xu , Y. Wang , J. Luo , D. Shi , W. Wu , L. Chen , Y. Jin , B. Jiang , C. Chen , Nat. Commun. 2025, 16, 160. 39747042 10.1038/s41467-024-55530-1 PMC11696470

[27] Z. Lei , B. Chen , Chem. Rev. 2017, 117, 6633. 28535681 10.1021/acs.chemrev.7b00246

[28] S. K. Singh , A. W. Savoy , J. Mol. Liq. 2020, 297, 112038.

[29] Y. Zheng , S. Zhang , Y. Yuan , C. Li , Adv Mater 2025, 37, 2503324. 10.1002/adma.202503324 40391621

[30] J. Zhu , Y. An , B. Xu , S. Mo , X. Zhou , Q. Zhang , Y. Wang , Y. He , Small 2025, 21, 2501621. 10.1002/smll.202501621 40343376

[31] R. C. Montoya , L. Álvarez de Cienfuegos , J. A. Gavira , J. W. Steed , Chem. Soc. Rev. 2024, 53, 10604. 39258871 10.1039/d4cs00271g

[32] S. J. K. O'Neill , M. Ashizawa , A. M. McLean , R. R. Serrano , T. Shimura , M. Agetsuma , M. Tsutsumi , T. Nemoto , C. D. J. Parmenter , J. A. McCune , G. G. Malliaras , N. Matsuhisa , O. A. Scherman , Adv Mater 2025, 37, 2415687. 40296300 10.1002/adma.202415687 PMC12232228

[33] S. Liu , Y. Li , J. Wen , Z. Shen , Q. Meng , Q. Liu , F. Yang , Z. Yu , J. Li , Z. Sun , G. Zhuang , J. Yang , Adv Funct Mater 2024, 34, 2313397.

[34] W. Yang , W. Zhong , S. Yan , S. Wang , C. Xuan , K. Zheng , J. Qiu , X. Shi , Adv Mater 2024, 36, 2312740. 10.1002/adma.202312740 38272455

[35] C. Chen , X. Yang , S. Li , C. Zhang , Y. Ma , Y. Ma , P. Gao , S. Gao , X. Huang , Green Chem. 2021, 23, 1794.

[36] Z. Wang , D. Chen , H. Wang , S. Bao , L. Lang , C. Cui , H. Song , J. Yang , W. Liu , Adv Mater 2024, 36, 2404297. 10.1002/adma.202404297 38734972

[37] X. Yang , B. Zhang , J. Li , M. Shen , H. Liu , X. Xu , S. Shang , Carbohydr. Polym. 2023, 313, 120813. 37182943 10.1016/j.carbpol.2023.120813

[38] R. a. Li , H. Zhang , L. Li , B. Zhang , X. Du , W. Shao , X. Qian , Y. Cao , Z. Liu , J. Mater. Chem. A. 2025, 13, 12988.

[39] H. Qiao , S. Sun , P. Wu , Adv Mater 2023, 35, 2300593. 10.1002/adma.202300593 36861380

[40] Y. Wang , K. Liu , W. Wei , H. Dai , Adv Funct Mater 2024, 34, 2402531.

[41] L. Yan , A. J. Huertas‐Alonso , H. Liu , L. Dai , C. Si , M. H. Sipponen , Chem. Soc. Rev. 2025, 54, 6634. 40491312 10.1039/d4cs01044b PMC12150016

[42] O. Hu , M. Lu , M. Cai , J. Liu , X. Qiu , C. Guo , C. Zhang , Y. Qian , Adv Mater 2024, 36, 2407129. 10.1002/adma.202407129 39073194

[43] W. Zhao , C. Wei , Y. Cui , J. Ye , B. He , X. Liu , J. Sun , Chem. Eng. J. 2022, 443, 136486.

[44] Y. Ma , Z. Zhao , Z. Zheng , J. Li , M. Li , J. Hu , Matter 2024, 7, 4046.

[45] C. Shi , X. Zhang , Q. Zhang , M. Chen , H. Tian , D. Qu , Chem. Sci. 2024, 15, 17460. 39371464 10.1039/d4sc05031b PMC11447730

[46] C. Dang , F. Zhang , Y. Li , Z. Jin , Y. Cheng , Y. Feng , X. Wang , C. Zhang , Y. Chen , C. Shao , Q. Zheng , H. Qi , Small 2022, 18, 2200421. 10.1002/smll.202200421 35426235

[47] C. Dang , M. Wang , J. Yu , Y. Chen , S. Zhou , X. Feng , D. Liu , H. Qi , Adv Funct Mater 2019, 29, 1902467.

[48] K. Hou , S. Zhao , D. Wang , P. Zhao , C. Li , J. Zuo , Adv Funct Mater 2021, 31, 2107006.

[49] C. Dang , F. Peng , H. Liu , X. Feng , Y. Liu , S. Hu , H. Qi , J. Mater. Chem. A. 2021, 9, 13115.

[50] A. Khan , R. R. Kisannagar , C. Gouda , D. Gupta , H. Lin , J. Mater. Chem. A. 2020, 8, 19954.

[51] X. Zhang , Q. Fu , Y. Wang , H. Zhao , S. Hao , C. Ma , F. Xu , J. Yang , Adv Funct Mater 2024, 34, 2307400.

[52] M. Chen , R. Yang , H. Wu , Q. Wang , C. Shi , S. Zhou , D. Yang , F. Liu , H. Tian , D. Qu , Angew. Chem. Int. Edit. 2024, 63, 202409200. 10.1002/anie.202409200 39031788

[53] Y. Zhang , Z. Zhao , L. Wang , F. Sun , J. Xu , B. Yao , Z. Sun , T. Liu , Y. Zeng , G. Zhang , W. Jiang , J. Fu , Adv Funct Mater 2025, e07942.

[54] B. Wang , Q. Zhang , Z. Wang , C. Shi , X. Gong , H. Tian , D. Qu , Angew. Chem. Int. Edit. 2023, 62, 202215329. 10.1002/anie.202215329 36602285

[55] D. Yang , K. Zhao , R. Yang , S. Zhou , M. Chen , H. Tian , D. Qu , Adv Mater 2024, 36, 2403880. 10.1002/adma.202403880 38723049

[56] Z. Ye , H. Yu , H. Xie , W. Zhu , S. Shi , C. Liu , Y. Wang , J. Liao , Q. Sun , D. Zhao , X. Shen , Adv. Sci. 2025, e06901. 10.1002/advs.202506901 PMC12463069 40559707

