# Supplementary material for: Supramolecular‐Engineered Bamboo Lignin Polymer with Enhanced Mechanical and Electrical Properties for Flexible Electronics
Source: Adv Sci (Weinh). 2025 Sep 12;12(43):e12983. doi: 10.1002/advs.202512983 (PMC12631916; doi:10.1002/advs.202512983)
Supplement: Supplementary file 1 — Supporting Information [file ADVS-12-e12983-s001.docx]

**Supplementary Information for**

**Supramolecular-Engineered Bamboo Lignin Polymer with Enhanced Mechanical and Electrical Properties for Flexible Electronics**

Zhimin Kou^1^, Ye Sha^2^, Lihong Hu^1^*, Meiting Liu^1^, Fuchun Huang^1^, Jie Wang^1^, Caiying Bo^1^, Yonghong Zhou^1^, Dawei Zhao^3^*, Puyou Jia^1^*

^1^Institute of Chemical Industry of Forest Products, Chinese Academy of Forestry (CAF),16 Suojin North Road, Nanjing 210042, China.

^2^Department of Chemistry and Material Science, College of Science, Nanjing Forestry University, Nanjing 210037, China.

^3^Key Laboratory on Resources Chemicals and Materials of Ministry of Education, Shenyang University of Chemical Technology, Shenyang, 110142, China.

*Corresponding to: [jiapuyou@icifp.cn;](mailto:jiapuyou@icifp.cn;) [daweizhao@syuct.edu.cn;](mailto:daweizhao@syuct.edu.cn;) hlh@icifp.cn

**I. Supplementary Method**

**Characterizations.** Fourier-transform infrared (FT-IR) spectra were obtained using a Nicolet iS50 FT-IR spectrometer (Thermo Scientific, USA) over the range of 4000-500 cm^-1^ at 4 cm^-1^ resolution. The Raman spectra were ac-quired using a DXR^TM^2xi Raman Imaging Microscope (Thermo Scienti-fic, USA) with a 785 nm excitation wavelength. The nuclear magnetic resonance (NMR) spectra of TA were obtained using a Bruker AV-400 spectrometer (Bruker Corporation, Germany) with deuterated chloroform (CDCl_3_) as the solvent and tet-ramethylsilane as the internal standard. X-ray photoelectron spectroscopy (XPS) was performed using a Kratos AxisUltra DLD X-ray photoelectron spectrometer (Kratos-Shimadzu Corpo-ration, Japan). The morphology of the freeze-dried hydrogel was observed using a scanning electron microscope (SEM, JSM-7600 Fs, JEOL, Japan) equipped with energy-dispersive spectroscopy (EDS).

**Rheology measurements**

The rheological tests were performed using a rheometer (HAAKE Mars 60; Thermo Fisher Scientific, Germany) equipped with a parallel plate (25 mm in diameter). The frequency sweep tests were conducted under a constant strain of 1 % from 0.1 to 100 rad·s^-1^ at room temperature. The strain sweep tests were performed at 25 °C and a constant frequency of 10 rad·s^-1^ within the range from 0.1 to 1000 %. The temperature sweep tests were measured from 5 to 120 °C with a heating rate of 3 °C/min under a constant strain of 1 % and a constant frequency of 10 rad·s^-1^. The step-strain tests were performed at small strain (1 %) and high strain (200 %) for brief intervals (5 min). During the measurements, both the storage modulus (G') and the loss modulus (G") were recorded.

**Mechanical properties**

The mechanical properties were evaluated using a universal testing machine (UTM6503, Shenzhen SANS, China) equipped with a load cell of 5kN. All the tests were performed three times and averaged to obtain the strength and strain. Rectangular hydrogels (30 mm length x 25 mm width x1.5 mm thickness) were used for tensile testing at a tensile rate of 100 mm/min, the elastic modulus was calculated from the slope of the initial linear region, The toughness was determined by integrating the area under the stress strain curves.

**Adhesive performance**

Lap shear measurements were performed to explore the adhesion capability of the hydrogels. The hydrogels were adhered to different substrates (PE, ceramic, glass, rubber, PTFE, and foam). The substrates were 100 mm length x 25 mm width. The adhered samples were placed in a stable environment for 12 h prior to testing. The samples were measured at a stretching speed of 20 mm/min. The measurements of all the samples were repeated three times, and the average adhesion strength was calculated.

**Self-healing and recycle:**

The hydrogels were cut into two halves with scissors and brought together immediately to stretch and light diodes to explore their self-healing performance. In addition, the recovery and reuse of the hydrogels were investigated through the gel-sol transitions. The formed hydrogel was cut into pieces, placed in a vial, and heated at 80 ^o^C for 15 min to convert the gel into a sol. The gel could be reformed as it was cooled to room temperature. The mechanical properties and ionic conductivities of the reformed hydrogels were recorded. The self-healing efficiency was calculated according to the following equation:

$$Self healing efficiency=\frac{S1}{S2}*100\%$$

where S_1_ and S_2_ represent the original stress and after healing stress of the hydrogel, respectively.

**Electrical measurements**

The ionic conductivity of the hydrogels was measured using linear current correction (LCR) digital electrical bridge (TH2830, Tonghui, China). The hydrogels were clamped with auxiliary copper sheets at both ends to connect them to the electrodes of an electrochemical work station, the ionic conductivity was calculated according to the following equation:

$$Conductivity=l/RS$$

where l, R, and S represent the effective length, bulk resistance, and cross-sectional area of the hydrogel, respectively.

The sensing performances of the hydrogels were measured using linear current correction (LCR) digital electrical bridge (TH2830, Tonghui, China). The change in resistance of the hydrogel during stretching was recorded to evaluate its sensing performance. The relative change in resistance was calculated using the following formula:

*ΔR/R =(R-R_0_)/R*

where R and R_o_ are the resistances after and before applying strain, respectively. The sensitivity of the flexible sensor was determined by gauge factor (GF), which was the ratio of the relative resistance change to the strain (*ε*) and obtained from the following equation,

*GF =(ΔR/R_0_)/ε*

Additionally, hydrogels adhere to different parts of the body. The change in resistance was recorded with the speech movement of the human body to explore its application in smart wearable materials.

**Statistical analysis:**

The statistical data were expressed as mean ± standard deviation. Each experiment was repeated at least three times to obtain the results. Statistical analyses were performed by one-way ANOVA with Origin 2021 statistical software.

**II. Supplementary Figures**

**Figure S1.** Synthetic route of PAL.

**Figure S2.** Synthetic route of PAL-Arg.

**Figure S3.** Synthetic route of *P*(LA-TA)-gel.

**
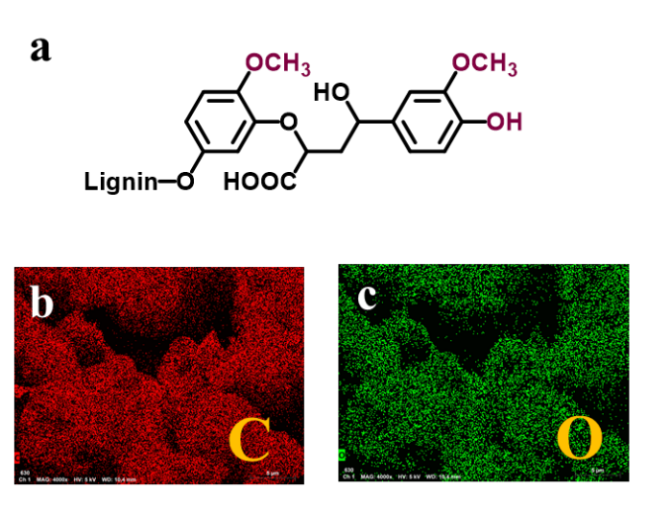
**

**Figure S4.** (a) The chemical structure of PAL. (b) C element mappings. (c) O element mappings.


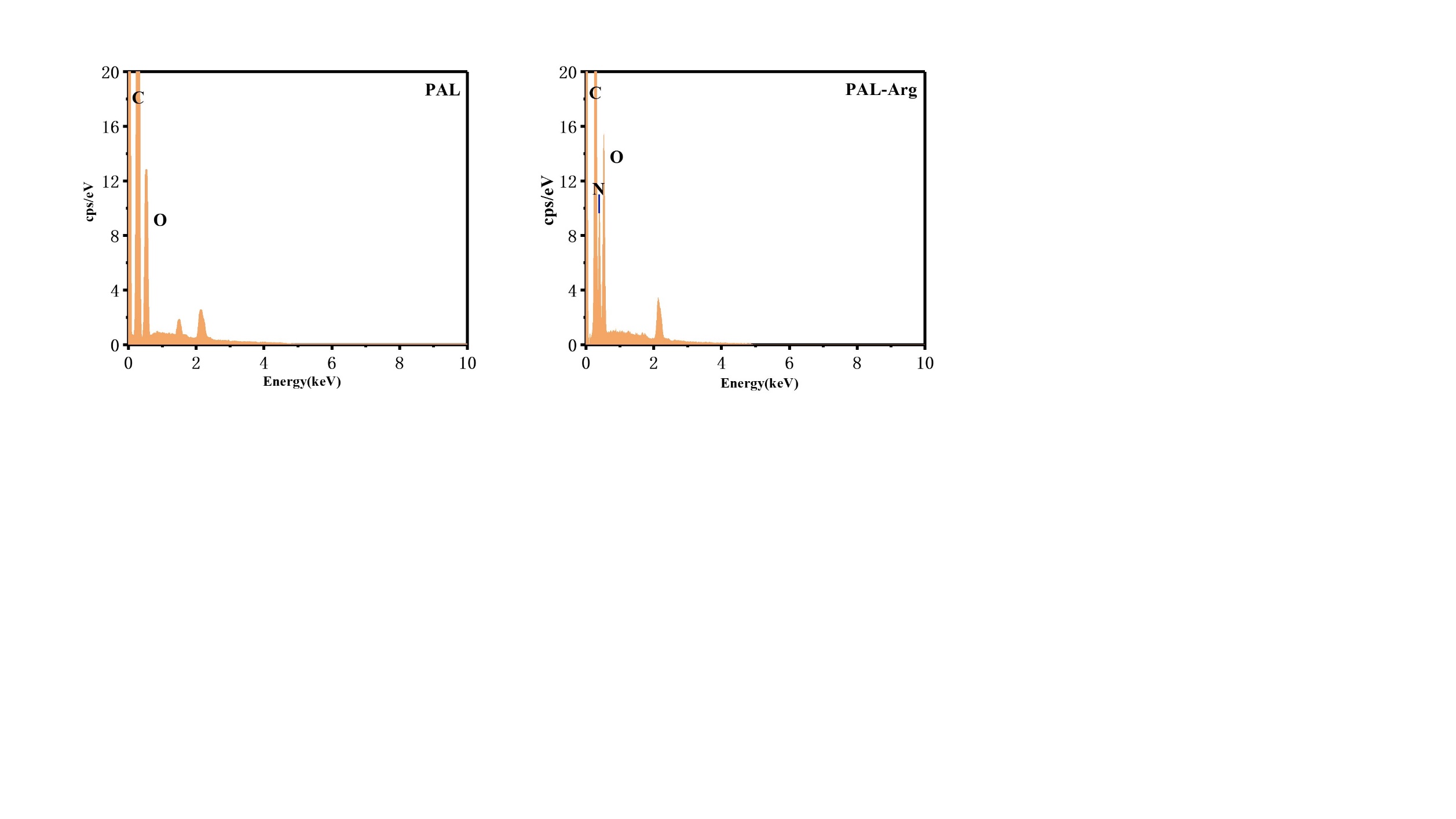


**Figure S5.** EDS mapping of PAL.

**
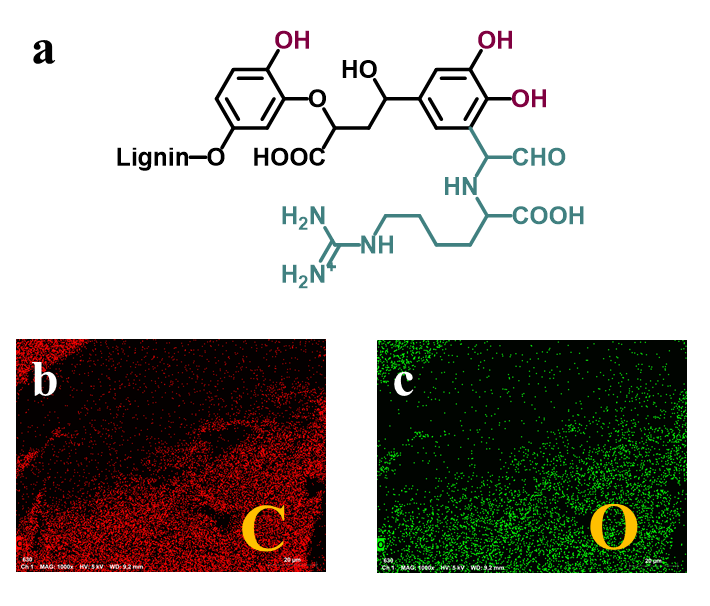
**


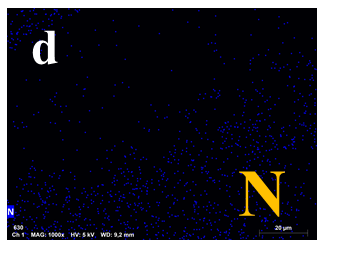


**Figure S6.** (a) The chemical structure of PAL-Arg. (b) C element mappings. (c) O element mappings. (d) N element mappings.

**
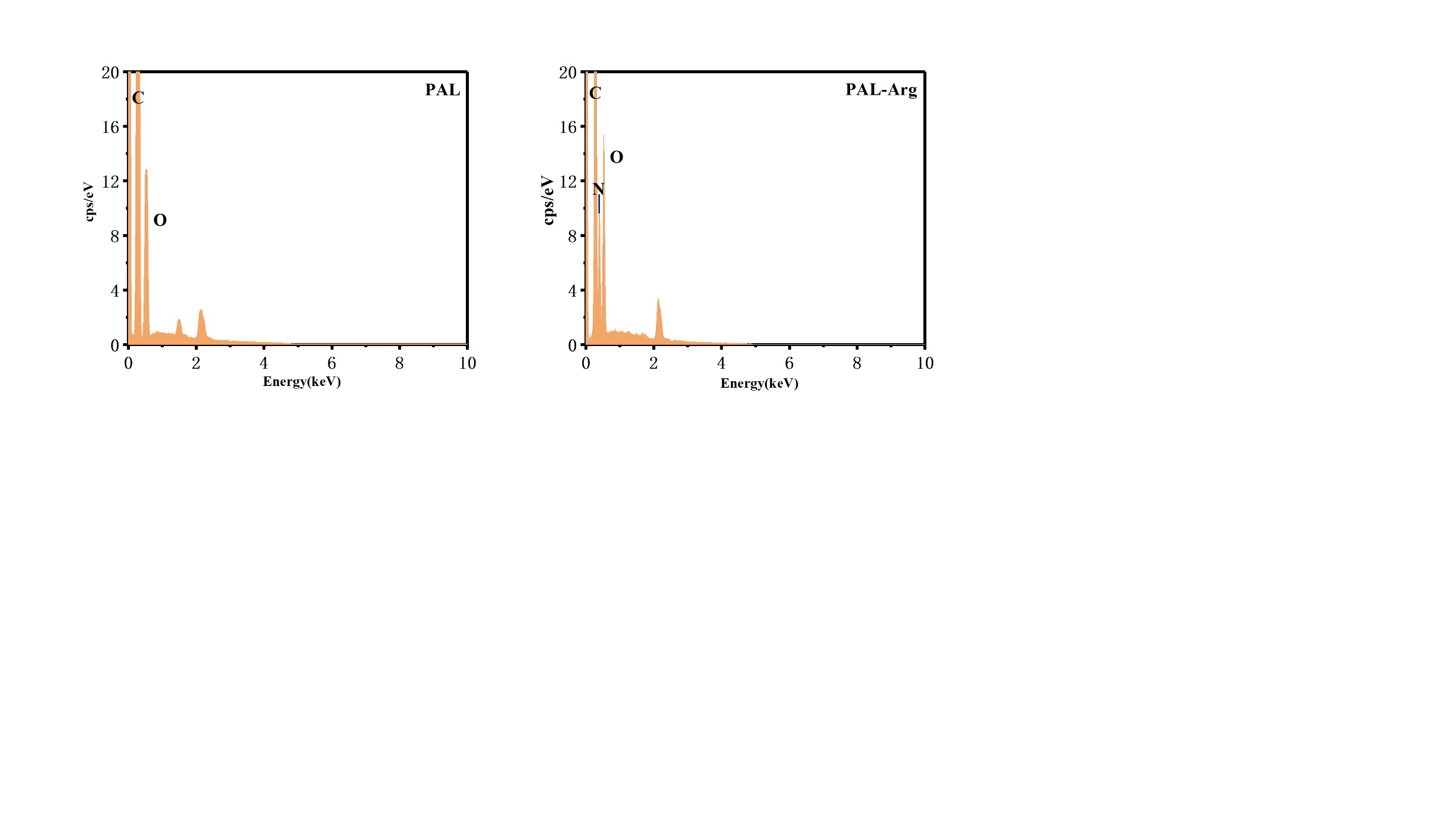
**

**Figure S7.** EDS mapping of PAL-Arg.


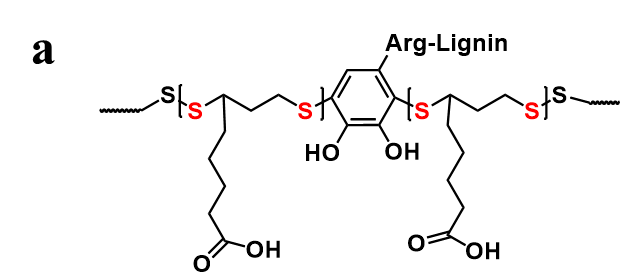


`
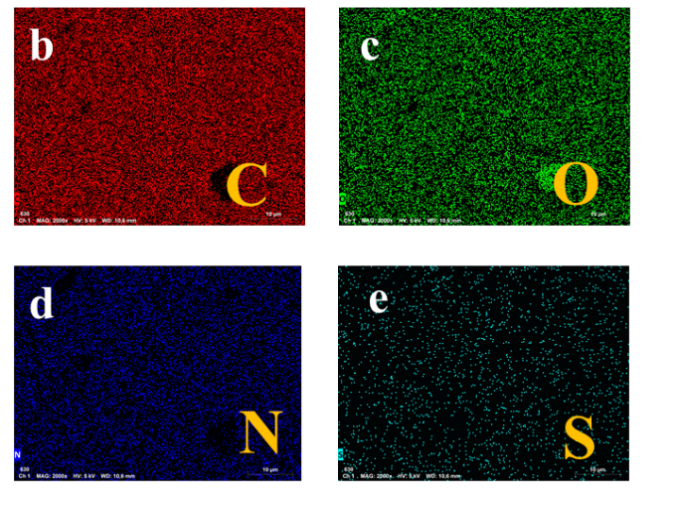


**Figure S8.** (a) The chemical structure of *P*(LA-TA)-gel. (b) C element mappings. (c) O element mappings. (d) N element mappings. (e) S element mappings.


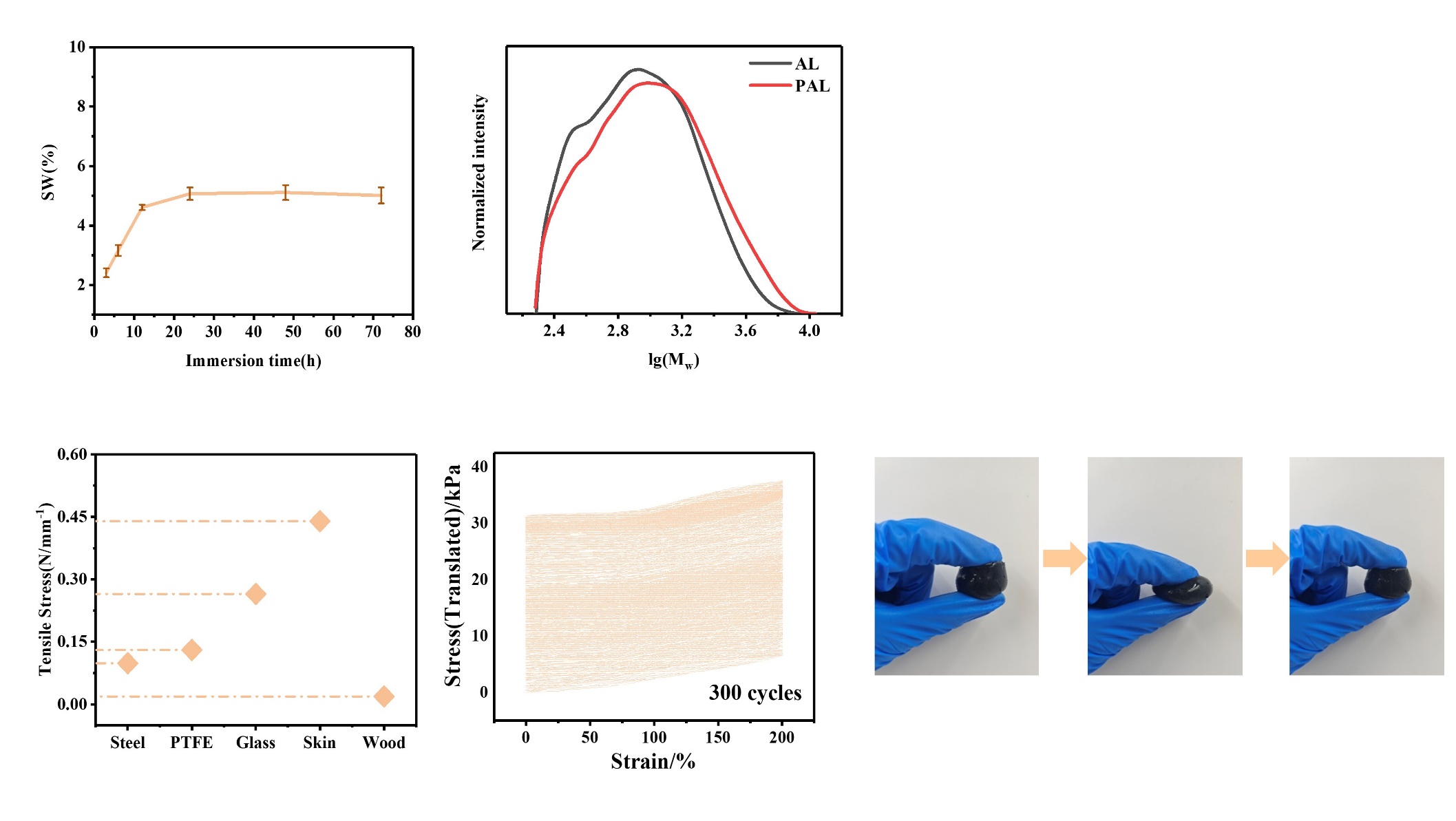


**Figure S9.** GPC of AL and PAL.


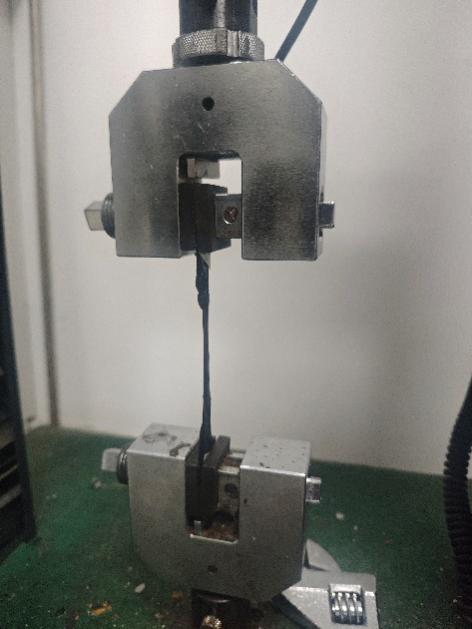

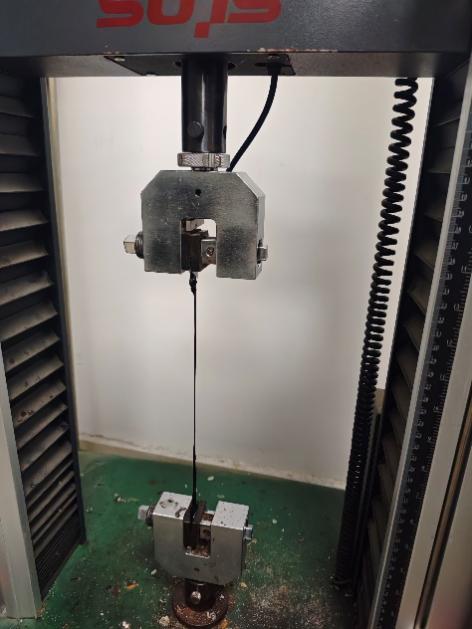


**Stretch**

**Figure S10.** Schematic diagram of the stretch of *P*(LA-TA)-gel.

**b**

**a**


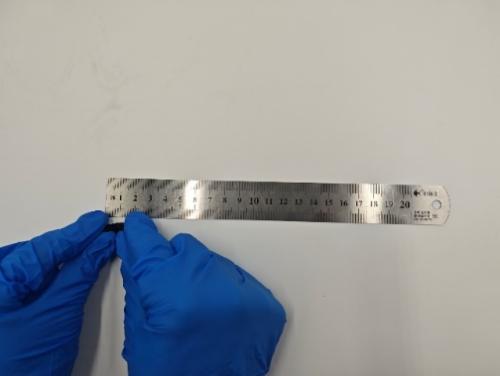

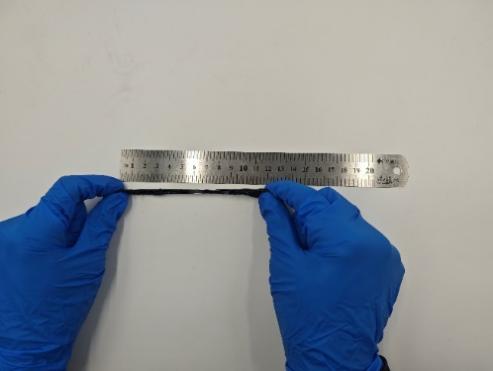


**Figure S11.** Tensile properties of *P*(LA-TA)-gel. Photos (a) before and (b) after stretching.


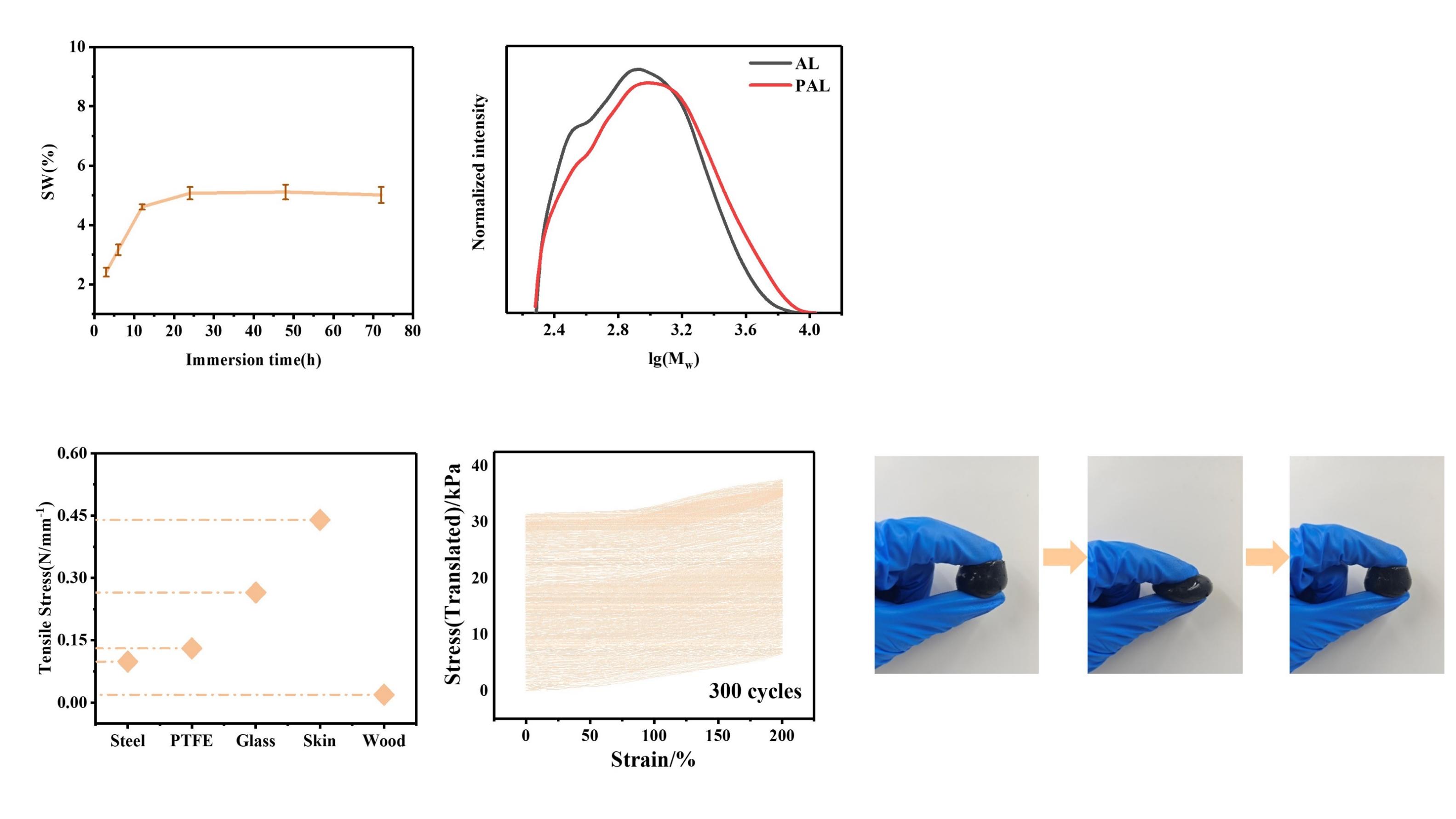


**Figure S12.** Squeeze ionic gel and its quick recovery of photos.


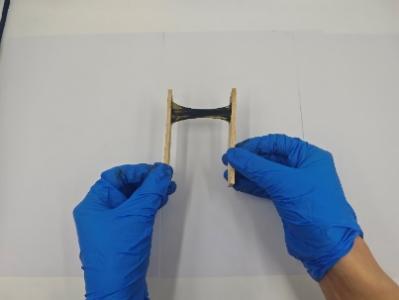
**
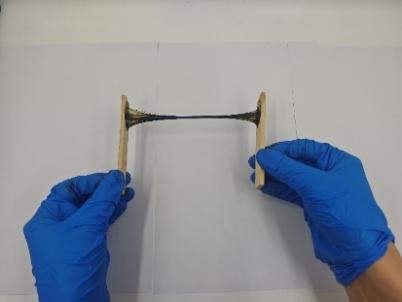

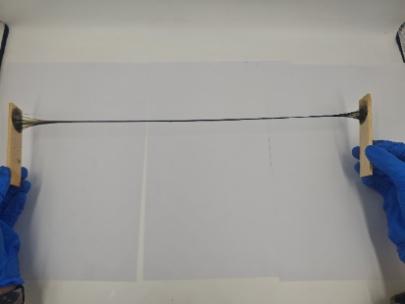
**

**c**

**b**

**a**

**Figure S13.** Adhesion Performance of *P*(LA-TA)-gel. (a)-(c) The state of the gel after the bonded wood chips are pulled apart.


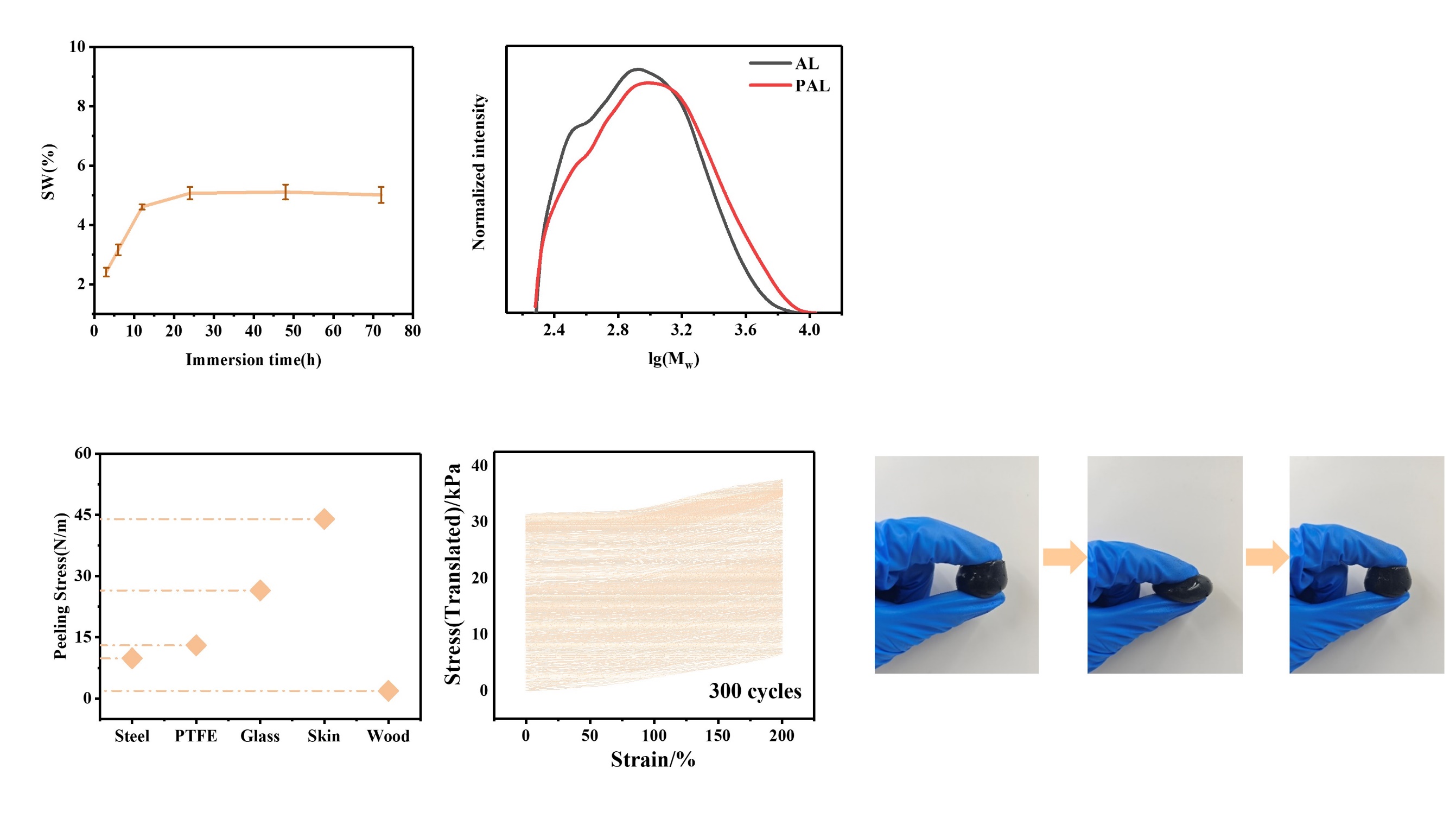


**Figure S14.** Peeling Strength of *P*(LA-TA)-gel.


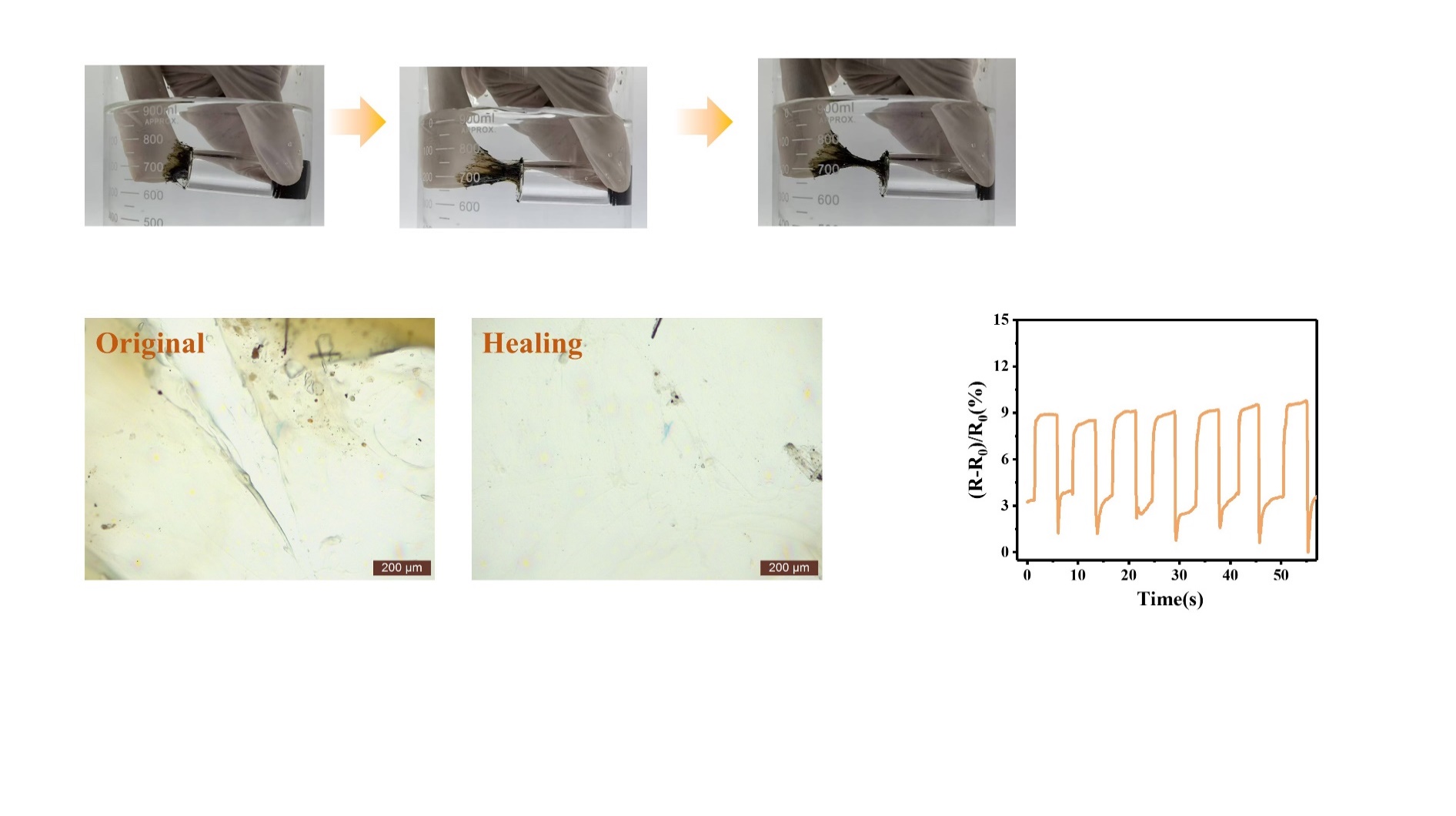


**Figure S15.** Underwater adhesion properties of *P*(LA-TA)-gel.

**a**

**b**

**c**


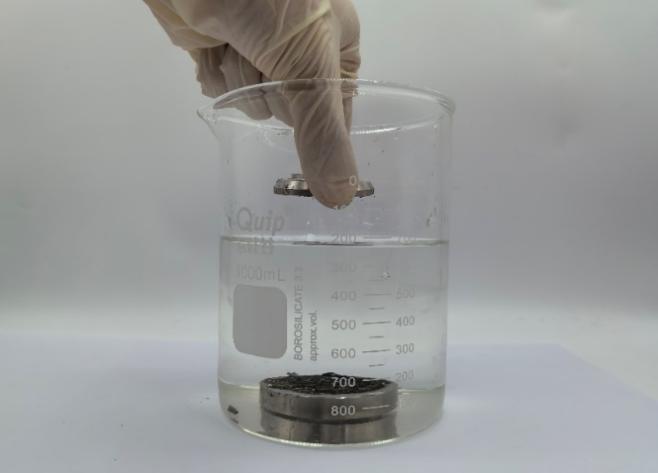

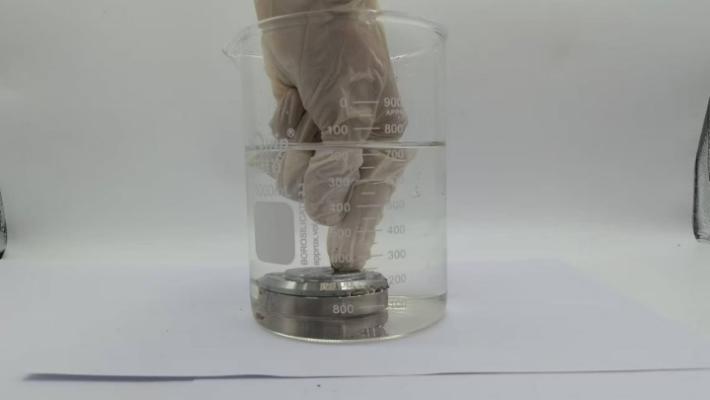

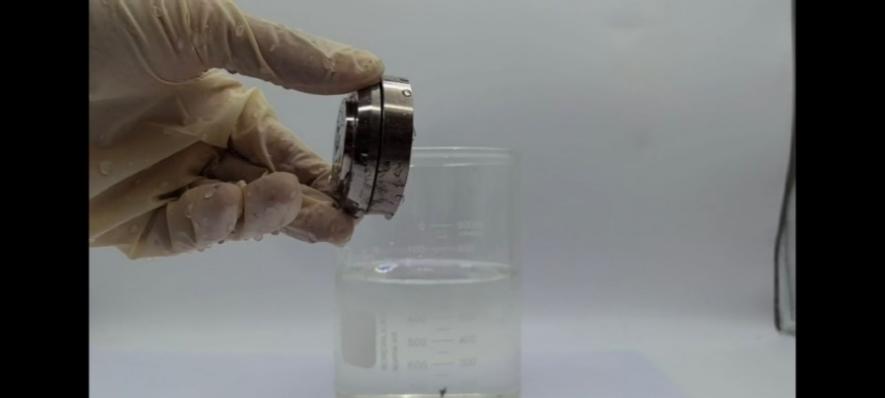


**Figure S16.** Underwater adhesion properties of *P*(LA-TA)-gel. (a)-(c) Photos of the *P*(LA-TA)-gel bonding iron blocks under water.


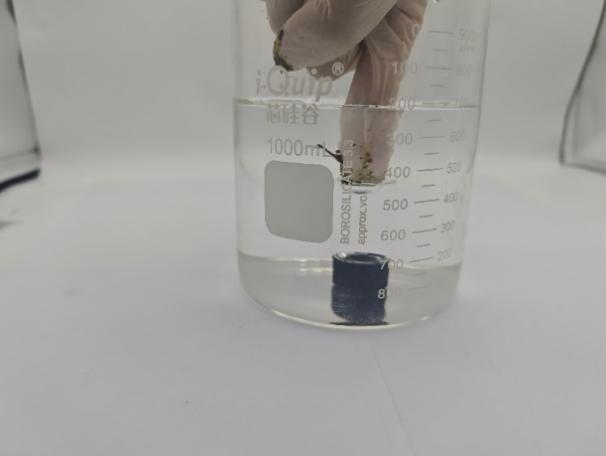

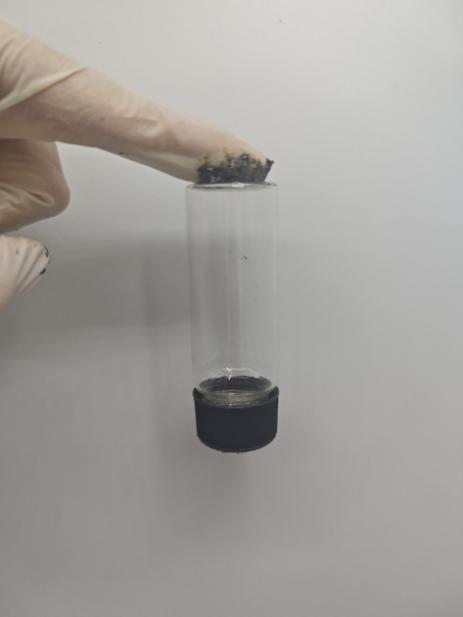


**b**

**a**

**Figure S17.** *P* (LA-TA)-gel has rapid underwater bonding properties. (a)-(b) Photos of the *P*(LA-TA)-gel bonding glass under water.


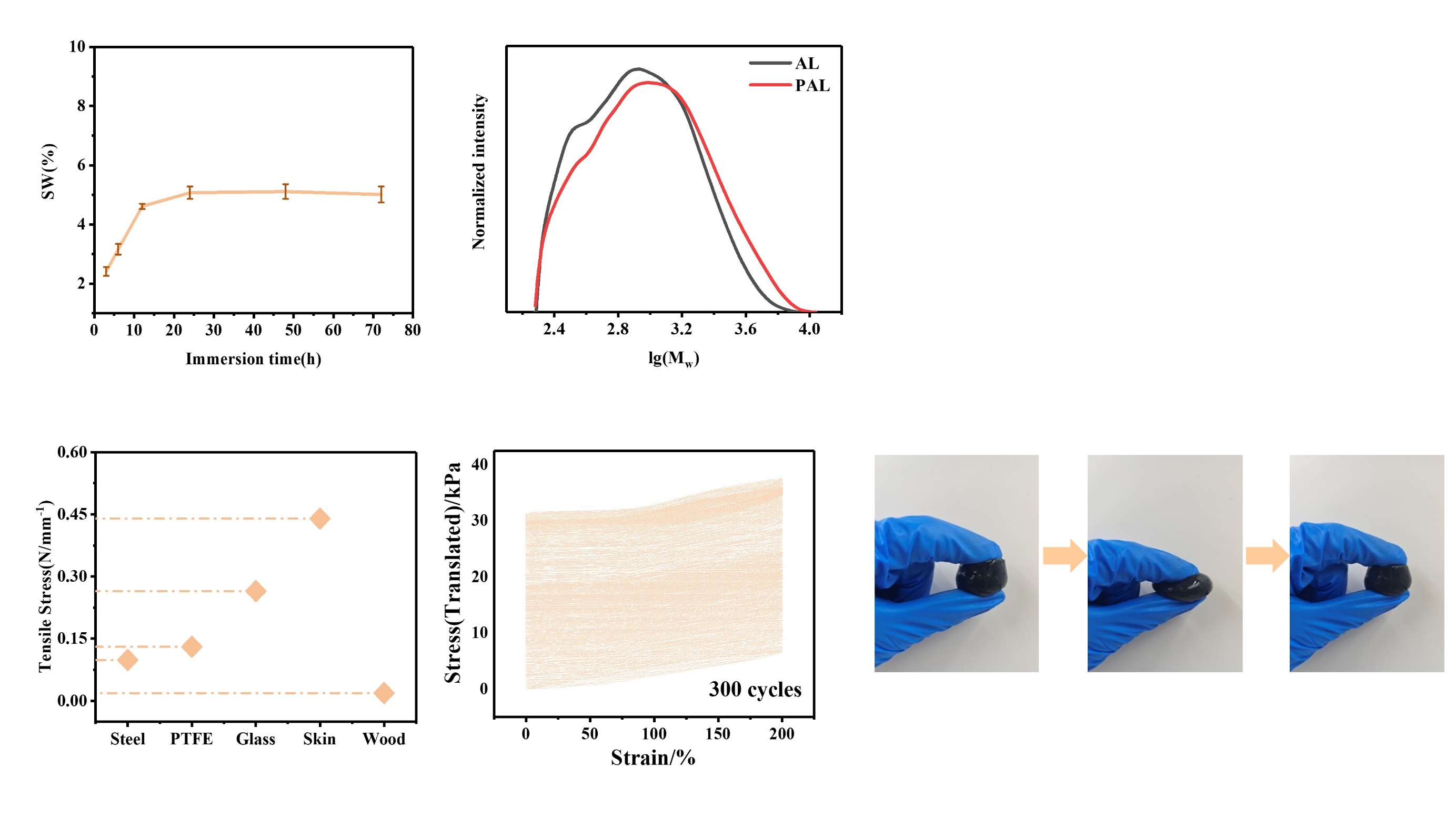


**Figure S18.** Swelling of *P*(LA-TA)-gel.


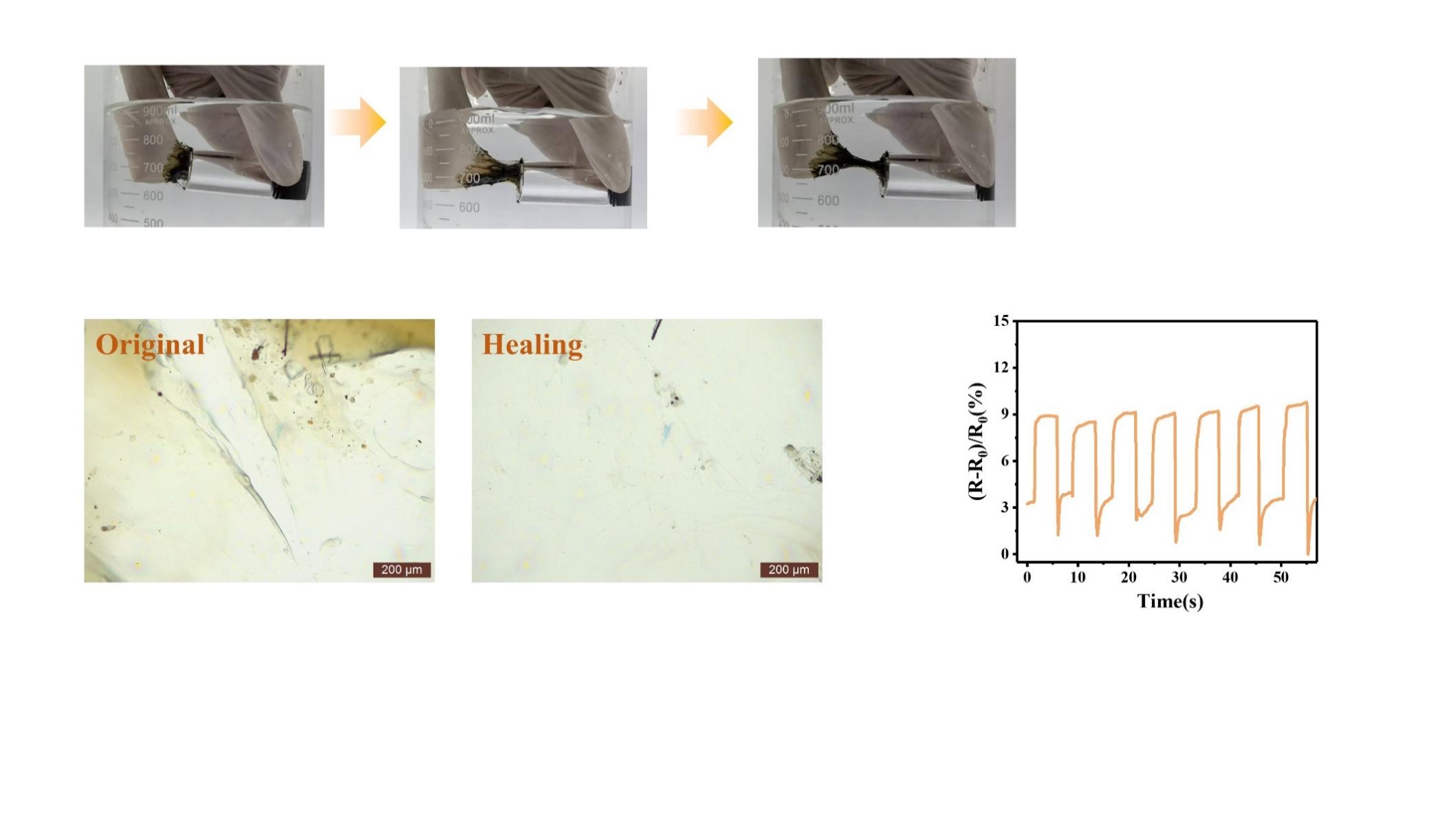


**Figure S19.** Microscope photographs of *P*(LA-TA)-gel before and after self-healing.


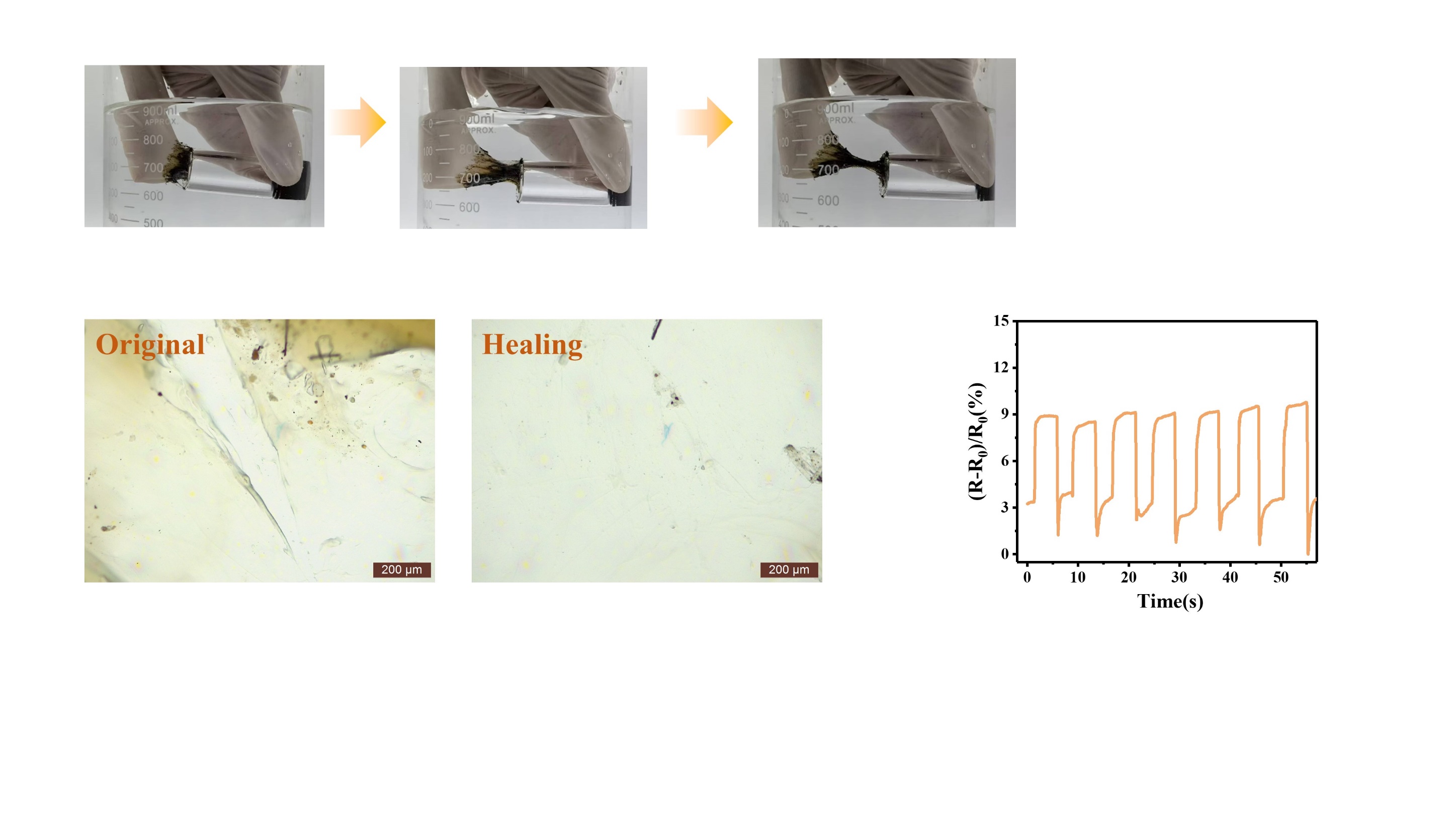


**Figure S20.** The *P*(LA-TA)-gel sensor responds to the change in relative resistance when the elbow is bent on the surface of human skin.

**Table S1.** Comparison of the *P*(LA-TA)-gel with the peer-reported conductive composites

|  | This Work | a | b | c | d | e | f |
| --- | --- | --- | --- | --- | --- | --- | --- |
| Strain/% | 1233 | - | 1100 | - | 800 | - | - |
| Self-healing efficiency | 90.5 | 82.7 | 90 | 97.1 | 80.1 | 80 | 52 |
| GF | 1.65 | - | - | 1.13 | 0.54 | - | - |
| Adhension  /kPa | 335.5 | - | 270 | - | 41 | 146 | 68.6 |
| Recyclability | Yes | Yes | - | - | Yes | - | - |
| Recovery rate/% | 100 | 80 | - | - | 75 | - | - |

**a:** LA-LiTFSl lonoelastomer **b:** LA-AA-CCl-FeCl_3_ lonogel

**c:** MASLA-PANI lonogel **d:** LA-TA-AlCl3-ILs lonoelastomer

**e:** LA-PA-PANI-FeCl_3_ lonogel **f:** TA@CNC-PAA-ChCl Elastomer

**Table S2.** Composition of the ionogels.

|  | TA/g | PAL/g | LiOH/g | H_2_O/g |
| --- | --- | --- | --- | --- |
| *P*(LA_3_-TA)-gel | 2 | 0.06 | 0.1 | 6 |
| *P*(LA_6_-TA)-gel | 2 | 0.12 | 0.1 | 6 |
| *P*(LA_9_-TA)-gel | 2 | 0.18 | 0.1 | 6 |
| *P*(LA_12_-TA)-gel | 2 | 0.24 | 0.1 | 6 |
| *P*(LA_15_-TA)-gel | 2 | 0.30 | 0.1 | 6 |
